# Endometrial extracellular matrix rigidity and IFNτ ensure the establishment of early pregnancy through activation of YAP

**DOI:** 10.1111/cpr.12976

**Published:** 2021-01-04

**Authors:** Tao Zhang, Shuai Guo, Han Zhou, Zhimin Wu, Junfeng Liu, Changwei Qiu, Ganzhen Deng

**Affiliations:** ^1^ Department of Clinical Veterinary Medicine College of Veterinary Medicine Huazhong Agricultural University Wuhan China; ^2^ College of Animal Science Tarim University Alar China

**Keywords:** endometrium, IFNτ, Mechanoresponses, pregnancy, YAP

## Abstract

**Background:**

In mammals, early pregnancy is a critical vulnerable period during which complications may arise, including pregnancy failure. Establishment of a maternal endometrial acceptance phenotype is a prerequisite for semiheterogeneous embryo implantation, comprising the rate‐limiting step of early pregnancy.

**Methods:**

Confocal fluorescence, immunohistochemistry and western blot for nuclear and cytoplasmic protein were used to examine the activation of yes‐associated protein (YAP) in uterine tissue and primary endometrial cells. The target binding between miR16a and YAP was verified by dual‐luciferase reporter gene assay. The mouse pregnancy model and pseudopregnancy model were used to investigate the role of YAP in the maternal uterus during early pregnancy in vivo.

**Results:**

We showed that YAP translocates into the nucleus in the endometrium of cattle and mice during early pregnancy. Mechanistically, YAP acts as a mediator of ECM rigidity and cell density, which requires the actomyosin cytoskeleton and is partially dependent on the Hippo pathway. Furthermore, we found that the soluble factor IFNτ, which is a ruminant pregnancy recognition factor, also induced activation of YAP by reducing the expression of miR‐16a.

**Conclusions:**

This study revealed that activation of YAP is necessary for early pregnancy in bovines because it induced cell proliferation and established an immunosuppressive local environment that allowed conceptus implantation into the uterine epithelium.

## Graphical abstract

In the endometrium of pregnant mammals, YAP activation promoted endometrial cell proliferation, induced EMT progression and supplied a weak inflammation benefit for uterus to receive the embryo. Mechanistically, expression levels of YAP were double regulated by upstream biochemical and mechanical signals in bEECs and partially dependent on the Hippo signalling pathway.

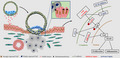

## 1 INTRODUCTION

Although advances in reproductive science over the past few decades have provided improved our knowledge of clinical conditions during pregnancy, the incidence of infertility and pregnancy complications has greatly increased worldwide.^1^, ^2^, ^3^ Many scientists have focused on elucidating the physiological mechanisms that occur during pregnancy, investigating the molecular expression network, and understanding space‐and time‐specific expression patterns to address reproductive diseases. However, our knowledge of embryos and the maternal uterus during early pregnancy in mammals remains limited, due to ethical considerations, technical limitations and high research costs.^4^, ^5^ Integrating the information gained from humans and other mammals during pregnancy is important for overcoming these difficulties.

In mammalian pregnancies, embryo implantation is a rate‐limiting step and critical vulnerable period during which complications arise and pregnancy failure occurs.^6^ A prerequisite for successful implantation is an intimate maternal‐foetal dialogue between the receptive uterine lining of the mother and the specialized trophectodermal cells of the embryo and this dialogue can be mediated by biochemical signals such as nucleic acids and hormones, as well as physical signals.^6^, ^7^, ^8^ It is widely accepted that soluble biochemical signals secreted by the conceptus, such as human chorionic gonadotropin (HCG) and IFNτ secreted by humans and ruminants, respectively, are required for pregnancy recognition.^9^, ^10^, ^11^ Well‐timed modification of mechanical parameters in the uterus during pregnancy is key to a successful pregnancy, including establishing an elastic fibre network, cell polarity, and matrix stiffness.^12^, ^13^, ^14^ Few studies have been performed on the molecular mechanistic changes caused by uterine mechanical behaviour during pregnancy. The response of the maternal uterus to the embryo is similar during early pregnancy in mammals, including decidualization and local immunosuppression. Therefore, further studies are necessary to uncover how the maternal uterus responds to biochemical and mechanical factors during early pregnancy, especially because this molecular mechanism may be evolutionarily conserved.

The Hippo pathway, which tightly regulates organ size and regeneration by restricting cell proliferation and stem cell self‐renewal, is an evolutionarily conserved protein kinase cascade in mammals.^15^, ^16^, ^17^ Recent findings indicate that the Hippo pathway is activated not only through soluble signals (eg, hormone and protein signals)^17^, ^18^ but also through physical and mechanical cues, such as cell‐cell junctions and the extracellular matrix (ECM).^18^, ^19^ The chemical and physical properties of hormonal and mechanical forces are integral to morphogenetic processes during embryonic development and endometrial decidualization, driving specific cell differentiation programs.^20^ To date, multiple studies on the Hippo pathway have focused on the differentiation of embryonic stem cells.^21^, ^22^ In contrast, little research has focused on whether and how the Hippo pathway controls the maternal uterus during early pregnancy. In this study, we report the identification of YAP in the maternal uterus as sensor and mediator of embryo signals that maintain early pregnancy.

## 2 MATERIALS AND METHODS

### 2.1 Reagents and plasmids

Bovine IFNτ and IL‐6 were purchased from Cloud Clone Corp (Wuhan, China). Verteporfin was purchased from Macklin Biochemical (Shanghai, China, V873898). Latrunculin A (Lat. A) was purchased from Santa Cruz Biotechnology (sc‐202691). Phalloidin was purchased from Sigma (P5282). miR‐16a mimics/inhibitors/NC and siYAP/LATS1/2/NC were generated by GenePharma (Shanghai, China). Their sequences are provided in Table S1. The reporter plasmids pGLO‐YAP‐3′UTR, pGLO‐YAP‐3′UTR‐MUT, pGLO‐NC (Promega) and the overexpression plasmid pcDNA3.1(+) –YAP were ordered from GeneCreate (Wuhan, China, Project Nos. 1 915 311, 1 915 312 and 1 916 340).

### 2.2 Bovines endometrial sample collection

Healthy cattle uteri were collected from a local commercial abattoir that had slaughtered cattle within the previous 30 min. The slaughterhouse is recognized by the Animal Welfare Organization and complies with the animal experiment rules of the Huazhong Agricultural University Animal Care and Use Committee. Non‐pregnant uteri (n = 10, oestrous) and uteri during early pregnancy (n = 25, 20‐60 d) were collected three times after washing with sterile PBS and then immediately transported to the laboratory on ice or fixed in 4% formaldehyde solution. Normal, healthy uterine horns were soaked in PBS containing 10% antibiotic/antimycotic solution after collection.

### 2.3 Bioinformatics analysis

Gene expression data and supplemental RNA‐seq data for endometria during pregnancy were downloaded from the GEO database (GSE107891). We obtained DEGs from the GEO database using the R package ‘edgeR’. Here, genes with an adjusted *P* value < .01 and a fold change (FC)>1.5 or <−1.5 were considered DEGs. In our study, the R package ‘clusterprofiler’ was used to provide functional classification of downregulated DEGs. We listed the top 10 terms in every category, and *P* < .05 was set as the cutoff point.

### 2.4 Primary culture

Bovine endometrial epithelial cells (bEECs) and endometrial stromal cells (bESCs) were isolated and cultured as previously described.^23^ In brief, uteri with no gross evidence of genital disease or microbial infection were soaked in PBS on ice for 1 hour until further processing in the laboratory. The tissue was digested in 20‐25 ml of DPBS (HyClone) sterile digestive solution, which contained 1% protease (Sigma, P5147) and 1% collagenase II (Sigma‐Aldrich). Following a 2 hour incubation in a shaking water bath at 37°C, the cell suspension was filtered through a 40‐μm mesh (Fisher Scientific, Loughborough, UK) to remove undigested material. The suspension was centrifuged at 1000 r/min for 5 minutes, and after three additional washes in washing medium, the cells were resuspended in DMEM/F‐12 culture medium containing 15% FBS (Gibco), 50 IU/ml penicillin, 50 IU/ml of streptomycin, and 2.5 μg/ml of amphotericin B (all from HyClone). The cells were cultured in 6 or 12‐well plates (Corning) or 25‐ or 75‐cm^2^ flasks at 37°C. The endometrial epithelial and stromal cell populations were isolated by differential adhesion to cell culture flasks. Immunofluorescence staining for the endometrial epithelial cell marker CK18 and the stromal cell vimentin marker was performed to detect cell purity. Primary cells were used in the next experiment after four passages.

### 2.5 Cell culture, transfections and treatments

Human HEK293 cells were purchased from American Tissue Culture Collection (ATCC). The cells were cultured under 5% CO_2_ in ambient O_2_ at 37°C in RPMI 1640 medium (Invitrogen) containing 10% foetal bovine serum (FBS, PAN, Germany).

2D culture on hydrogels with high (40 kPa) or low (1 kPa) stiffness was performed as described elsewhere.^24^ In brief, 10 μg/ml bovine fibrinogen (Solarbio) was used to coat hydrogels activated with Sulfo‐SANPAH (Thermo Fisher Scientific) according to the needs of the cells.

bEECs at 60% confluence were exposed to 0.5 μM Lat. A, 10, 50, or 100 ng/ml IFNτ or an equal volume of PBS for 24 hour and then cells were collected and stored at −20°C for the next experiment. Transient transfection of HEK293 and primary cells was carried out using Lipofectamine 2000 (Invitrogen) as recommended by the manufacturer. Briefly, Lipofectamine 2000 and the plasmids, siRNA or miR‐16a mimics/inhibitor/NC were separately dissolved in 500 μl of Opti‐MEM I medium (Gibco), and then these solutions were mixed and incubated for 20 min at room temperature to form complexes. The prepared mixture was added to exponentially growing cells (4 × 10^5^) seeded in a 6‐well plate for 12 hours. All transfections were performed in triplicate, and the cells were cultured in OPTI‐MEM medium for 24 hour for RNA studies, or 48 hour for protein studies.

### 2.6 Mice and treatment

Female (aged 6‐8 weeks) and male (aged 8‐10 weeks) Kunming (KM) mice were purchased from the Laboratory Animal Service Centre of Huazhong Agricultural University. All mouse experiments were conducted in accordance with the university guidelines on animal experimentation (HZAUMO‐2015‐12), and approval by the Animal Ethics Committee of Huazhong Agricultural University was obtained for all related procedures. The mice were maintained at room temperature with a 12‐h dark‐light cycles and free access to food and water.

For the normal early pregnancy model, sexually mature female KM mice aged 6‐7 weeks were randomly paired with a KM male mouse aged 8‐10 weeks at 7:00 PM, and female mice that did not mate during the oestrous period were used as the non‐pregnant group. The day a vaginal plug was observed the next morning after mating, at 8:00 AM was designated day 0.5 of pregnancy (ED 0.5). Uterine tissue was collected from normal pregnant mice at ED 0.5, ED 2.5, ED 4.5 and ED 8.5. For the pregnancy interference model, on ED 1.5, mice were intraperitoneally injected with verteporfin (VP, 10 μmol/kg) and an equal amount of PBS (control) every two days or on ED 2.5, mice were intrauterine injection with 50μL siYAP/siNC (30 pmol) combined with Lipofectamine. Mice were euthanized on ED 7.5, and the uterus was imaged and weighed immediately. For intrauterine injection of the IFN‐τ model described previously, female mice were randomly divided into two groups: (1) control and (2) IFN‐τ, in which IFN‐τ (10 mg/kg, 20 μl) was intrauterine injected into mice every two days. The control group was injected with an equal volume of PBS. The mouse uterus was collected on the seventh day for further analysis. Then the uterus was flash frozen in liquid nitrogen and stored at −80°C or fixed in 4% formaldehyde for subsequent analysis. All experimental and control groups contained 5 to 10 mice each.

The oil‐induced decidualization mouse model was adapted as previously reported.^3^ Pseudopregnancy was induced in sexually mature females that were each randomly paired with vasectomized males, and the day a vaginal plug was observed was designated as day 0.5 of pseudopregnancy. Intrauterine injection of 25 μl oil into one uterine horn was performed on day 3.5 of pseudopregnancy, and the contralateral uninjected horn served as a control (n = 6). The uterus was photographed and weighed on day 7.5.

### 2.7 Dual‐luciferase assays

293T cells were plated onto 6‐well plates at a density of 4 × 10^5^ cells/ml per well and cotransfected with pGLO‐YAP or pGLO‐YAP MUT and miR‐16a mimics using Lipofectamine 2000. The luciferase activity was assessed using the Dual‐Glo luciferase assay kit (Promega), according to the manufacturer's protocol. The cells were then lysed to measure luciferase activity according to the manufacturer's instructions.

### 2.8 Cell proliferation assay

Cell proliferation was evaluated using CCK‐8 (Dojindo Laboratories) assays according to the manufacturer's instructions. In brief, cells were seeded in 96‐well plates at a density of 3 × 10^4^ cells/ml, and five repetitions were performed for each group. After treatment, the cells were continuously cultured with 10 μL of CCK‐8 in each well at 37°C for 2 hours. Cell proliferation was measured through absorbance (optical density) at 450 nm with a microplate reader (Bio‐Rad Instruments).

### 2.9 Gene expression analysis by RT‐qPCR

Total RNA was isolated using TRIzol® reagent (Invitrogen) and cDNA was synthesized using the HiScript® II Q Select RT SuperMix for qPCR kit (Vazyme Biotech Co., Ltd). Quantitative RT–qPCR was performed in duplicate using FastStart Universal SYBR Green Master Mix (Roche Applied Science) using the StepOne real‐time PCR System (Life Technologies Corp.). The expression levels of miRNA were assessed using a Hairpin‐itTM microRNA qPCR Quantitation Kit (GenePharma) according to the standard protocol. Expression levels are given relative to GAPDH or U6. Sequences of primers are provided in Tables S2 and S3.

### 2.10 Total, nuclear and cytoplasmic protein extraction

Total protein from cells or tissue was extracted according to the protein extraction kit (Vazyme). Nuclear and cytoplasmic proteins were extracted according to the Nuclear and Cytoplasmic Protein Extraction Kit (Sangon Biotech). The protein concentration was determined using a BCA protein assay kit (Va6zyme). β‐actin and PARP were used to assess the purity of the cytoplasmic fraction and the nuclear fraction respectively.

### 2.11 Western blot analysis

The protein was separated by SDS–PAGE (5%‐12%), transferred onto 0.45 μm PVDF membranes (Solarbio) and blocked in 10% non‐fat milk in TBST for 2 hour at room temperature. The blots were successively incubated with primary antibodies overnight at 4°C. Following three washes with TBST, the membranes were then incubated with secondary antibodies at room temperature for 2 hour. After three washes, the membranes were subjected to chemiluminescence using Clarity Western ECL Substrate (Affinity). Anti‐β‐actin was used as control. Protein expression was detected using an enhanced chemiluminescence detection system (ImageQuant LAS 4000 mini, USA). Antibody information is provided in Table S4.

### 2.12 Immunofluorescence

Cells grown on glass coverslips were fixed with 4% paraformaldehyde for 15 min at room temperature, washed three times with PBS and then permeabilized with 0.05% Triton X‐100 for 10 min at room temperature. Following three 5 min washes in PBS, the coverslips were blocked with 5% BSA for 30 min and then incubated overnight with antibodies against YAP, E‐cadherin and Ki67 at 4°C. Following three 5 min washes in PBST, the coverslips were incubated with secondary antibodies for 1 hour at room temperature in the dark. Following three 5 min washes with PBST, the coverslips were stained with DAPI. Images were obtained using an Imager Nikon Eclipse C1 (Japan), a Nikon DS‐U3 (Carl Zeiss) or an optical microscope (Olympus).

### 2.13 Histology and immunohistochemical analysis

Maternal uteruses were extracted from pregnant female cows or mice. The uterus was fixed in 4% formaldehyde solution and the tissue was embedded in paraffin. Sections (4 mm) were cut and stained with haematoxylin and eosin. Immunohistochemistry was performed on 4 micron‐thick, FFPE sections using an anti‐YAP antibody. FFPE sections were deparaffinized using xylene and rehydrated in graded ethanol. Sections were treated in 1 mM EDTA, pH 8.0 by boiling at 125°C for 30 seconds and 90°C for 10 seconds inside a cocker within a microwave for antigen retrieval. All sections were incubated with endogenous peroxidase with 1% H_2_O_2_ for 10 minutes and protein blocking reagents for 5 minutes each. The sections were then incubated with YAP antibody diluted in TBST diluent for 1 hour at room temperature. Following primary antibody incubation, the sections were incubated with monoclonal mouse anti‐rabbit immunoglobulins for 1 hour at room temperature. Afterwards, the sections were washed with PBS and then counterstained with haematoxylin. Images were obtained using an Imager Nikon Eclipse C1, a Nikon DS‐U3 (Carl Zeiss) or an optical microscope (Olympus).

### 2.14 Statistics and reproducibility

The in vitro experiments were repeated at least three times unless stated otherwise. As indicated in the figure legends, all quantitative data are presented as the mean ± SD or mean ± SEM of three biologically independent experiments or samples. Statistical analyses were performed using GraphPad Prism 8 and Excel. Statistical significance was tested using an unpaired Student's t test or one/two‐way ANOVA with Sidak's multiple‐comparisons test. ANOVA was used to compare more than two groups. Exact *P*‐values are included in Table S5. All data were considered statistically significant at *P < .05, **P < .01.

## 3 RESULTS

### 3.1 YAP is activated in the normal bovine endometrium during early pregnancy

**FIGURE 1:**
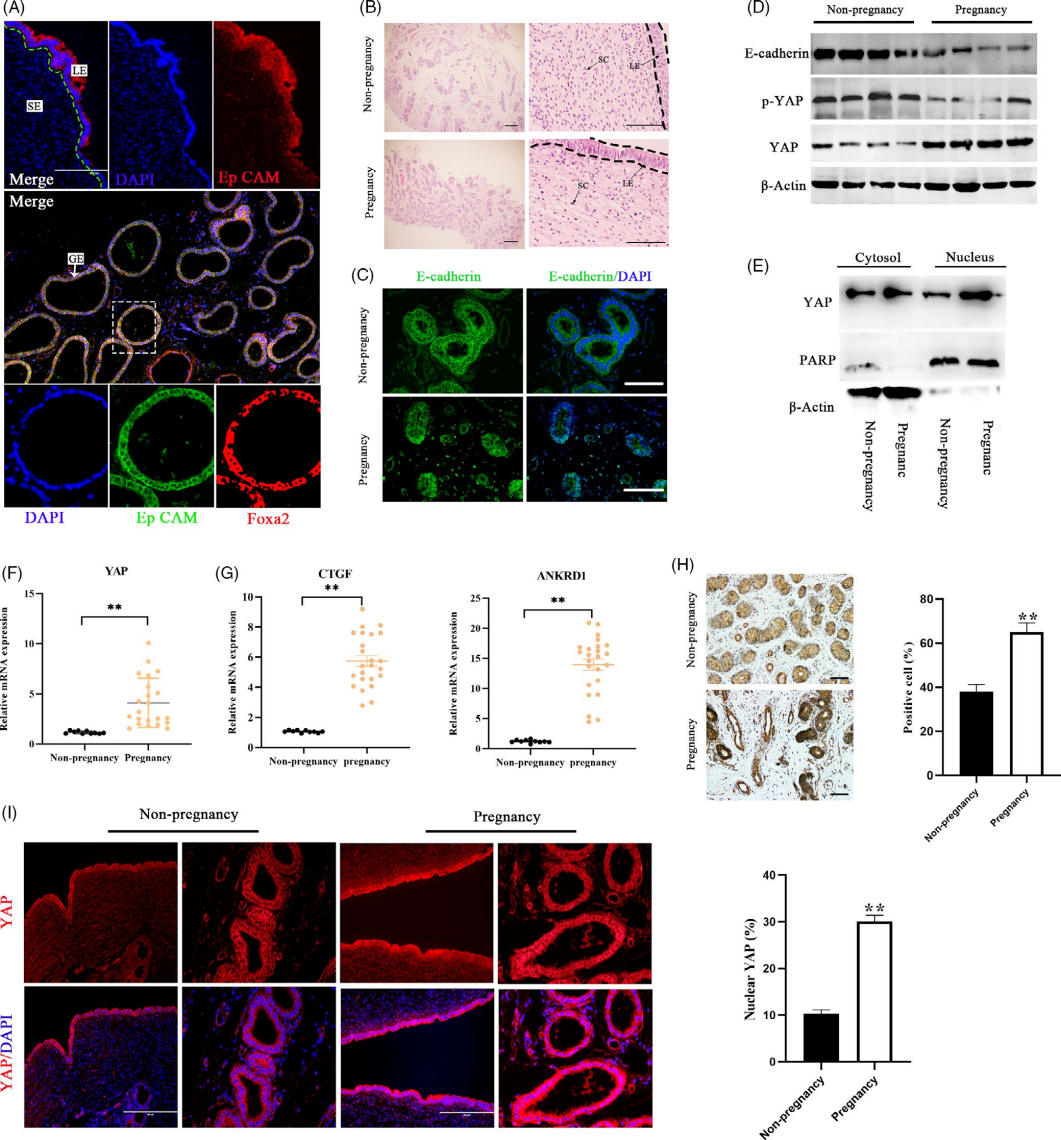
YAP is activated in the endometrium of pregnant bovines. A, Representative bovine uterine epithelia labelled with EpCAM antibody (epithelial marker), FOXA2 antibody (glandular‐specific marker), and DAPI (nuclei) in a uterine tissue section. Higher magnification of the glandular tissue indicated in the dotted square in the lower merge panel is shown in the lower panel. Scale bars: 200 μm (up), 400 μm (medium) and 20 μm (lower). B, Representative histopathological images from uterine tissue sections in pregnant and non‐pregnant cows. Scale bars: 400 (Left) and 50 μm (Right). C, E‐cadherin visualized by immunostaining (green); nuclei were counterstained with DAPI (blue), n = 2. Scale bars, 50 μm. D, Protein expression of E‐cadherin, YAP and p‐YAP in bovine uterine tissue by western blot, n = 3. E, Western blotting for YAP in nuclear and cytoplasmic protein fractions from bovine endometrium, n = 3. F, G, mRNA levels of YAP (f), CTGF and ANKRD1(g) in bovine endometrium were detected by RT‐qPCR, n = 3. H, Representative immunohistochemical images (left, n = 2) and quantification of YAP positive cells (right, n = 3). Scale bars 200 μm. I, Representative immunofluorescence (n = 2) and quantifications of nuclear and cytoplasmic subcellular localization of YAP (right, n = 15) in bovine endometrial epithelial cells. Scale bars 100 μm. Experiments were repeated *n* times with two biological replicates. Data are shown as the mean ± SEM. *P* values were determined by an unpaired two‐sided t test. **P < .001. See also Figure S1

To gain insight into transcriptional regulation during early pregnancy, we examined RNA‐sequencing data^25^ and microarray data from the GEO database (GSE107891). Consistent with previous analyses,^25^, ^26^ genes enriched in gene ontology (GO) terms for cellular component, ECM and cell adhesion were downregulated in pregnant cows compared to non‐pregnant cows (Figure S1). In bovines, the uterine endometrium is primarily composed of stromal cells and luminal (marked by EpCAM) and gland epithelial cells (marked by Foxa2) (Figure 1A). In the cohort of samples of bovine endometrium from successful pregnancies (n = 25) and non‐pregnancy uteri in oestrus (n = 10), we confirmed this phenomenon. The results of histopathology experiments showed that the number of glands and the gap between endometrial cells increased in cattle early pregnant (Figure 1A,B). The expression of E‐cadherin was markedly decreased in the pregnancy group, indicating weakened cell adhesion junctions (Figure 1C,D). These results are consistent with the previously reported E‐cadherin mediated regulation of epithelial cell polarity loss, ensuring embryo attachment.^27^ Therefore, we speculated that YAP is functionally required for successful pregnancy, and is a sensor and mediator of the structural and mechanical features of the cellular microenvironment. We found that the mRNA and protein levels of YAP were significantly higher in the pregnant group than in the non‐pregnant group and that the phosphorylation levels of YAP were significantly decreased (Figure 1D‐F). We also assayed two of the best YAP regulated genes from our signature, CTGF and ANKRD1, and the results showed that their expression levels were high in the uterus of pregnant cows (Figure 1G). In addition, we found that YAP was clearly cytoplasmic in the pregnant endometrium but became predominantly nuclear in the non‐pregnant endometrium, and YAP expression in endometrial epithelial cells was higher than that in stromal cells (Figure 1H,I). These findings prompted us to investigate whether YAP activation in the endometrium may regulate pregnancy.

### 3.2 YAP is regulated by cell spreading and ECM stiffness in bovine endometrial cells

**FIGURE 2:**
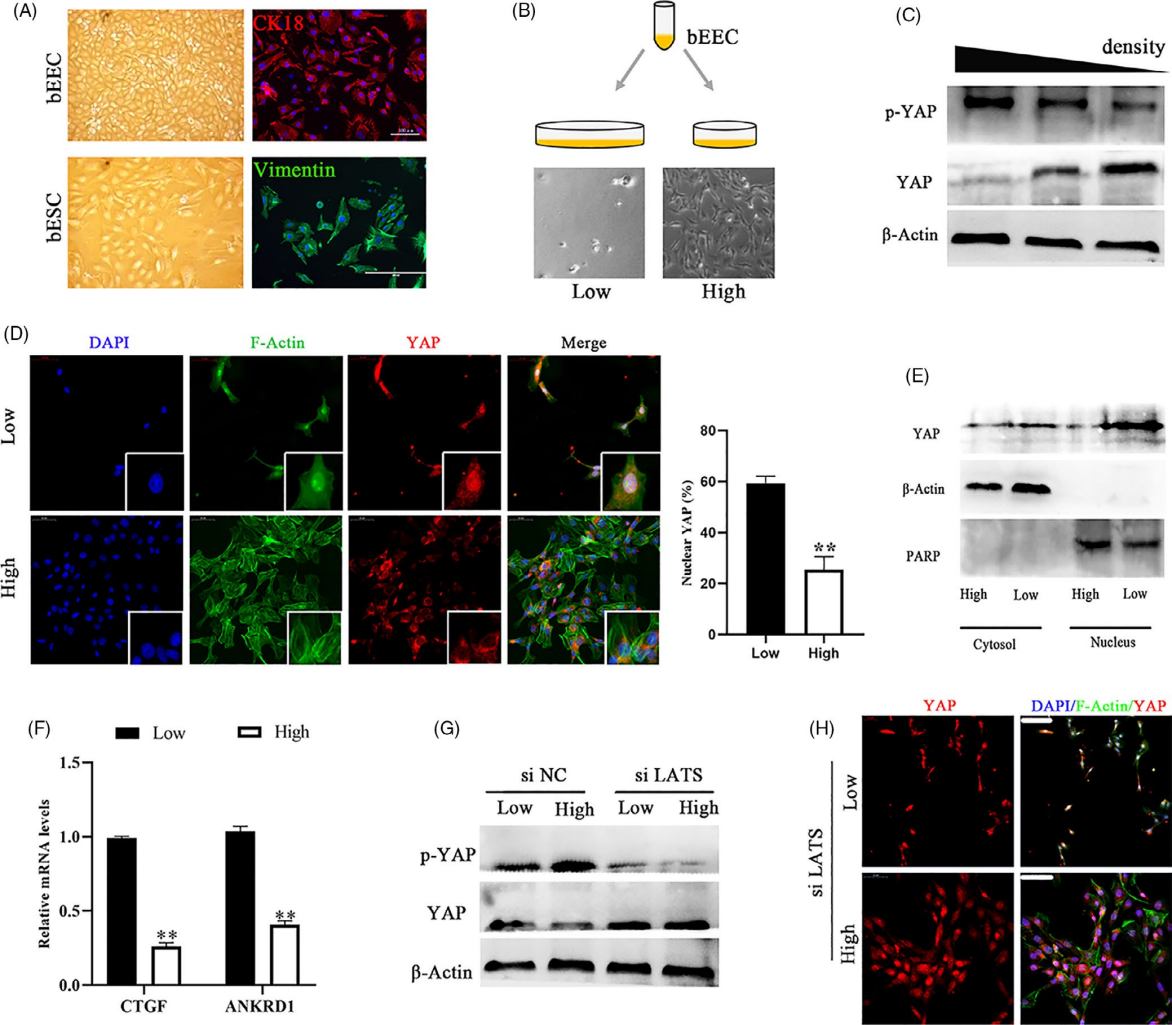
YAP is regulated by cell density in a manner dependent on the Hippo pathway. A, Images of primary cultured bovine endometrial cells (left) and immunofluorescence images corresponding to cell type markers (right). bEECs, bovine endometrial epithelial cells, CK18; bESCs, bovine endometrial stromal cells, vimentin. Scale bars, 100 μm and 50 μm, n = 3. B, Schematic representation of different bEEC density culture methods and images of actual effects. The same number of cells were seeded in culture plates of different sizes. C, Protein expression of YAP and p‐YAP in bEECs at different cell densities, n = 3. D, Immunofluorescence images (left, n = 2) and quantification of the nuclear and cytoplasmic subcellular localization of YAP (right, n = 15) in bEECs, when grown at low/high densities. Scale bars, 50 μm. Enlarged = 10 μm. E, YAP expression in the cytosol and/or nucleus of bEECs grown at low/high density, n = 3. F, mRNA levels of CTGF and ANKRD1 in bEECs cultured at low/high density were detected by RT‐qPCR, n = 3. G, Protein expression of YAP in bEECs treated with siNC or siLATS at low/high density, n = 3. H, Representative immunofluorescence of YAP in bEECs treated with siLATS at low/high densities, n = 2. Scale bars, 50 μm. DAPI, blue, nuclei; F‐actin, green, cell boundaries; YAP, red. All experiments were repeated independently three times with similar results, n = 3. Experiments were repeated *n* times with two biological replicates. Data are shown as the mean ± SEM. *P* values were determined by an unpaired two‐sided t test. **P < .001. See also Figure S2A–C

To investigate the cross‐talk between YAP and mechanical cues in the bovine endometrium during pregnancy, primary epithelial and stromal cells were isolated according to previously described methods.^28^ The stromal cell marker vimentin and epithelial cell marker keratin 18 (CK18) were used to determine the purity of isolated cells, and when the cell purity was 95% or higher, the cells were used for further experiments (Figure 2A). A previous study reported that YAP activity is affected by cell density in cancer cells^29^; therefore, we hypothesized that the low cell density of bEECs induced YAP activation. When bEECs were grown at low cell density (<40% confluent), YAP became dephosphorylated and translocated into the nucleus compared to cells grown at high density (>80% confluent) (Figure 2B‐E). We also found that the expression of the YAP target genes CTGF and ANKRD1 was repressed by growth at low density in bEECs (Figure 2F). In addition, cell density obviously affected YAP phosphorylation on serine 127 (Figure 2C), and we speculated that cell density mediated YAP activation was dependent on the Hippo signalling pathway. Then we knocked down LATS1/2, which is a core protein kinase of the Hippo pathway that induces phosphorylation of YAP, by siRNA interference (Figure S2A,B). The results suggested that knockdown of LATS1/2 can rescue the expression of YAP in cells at high confluence (Figure 2G,H, Figure S2C). Thus, cell crowding controls YAP in a manner largely dependent on the Hippo signalling pathway.

**FIGURE 3:**
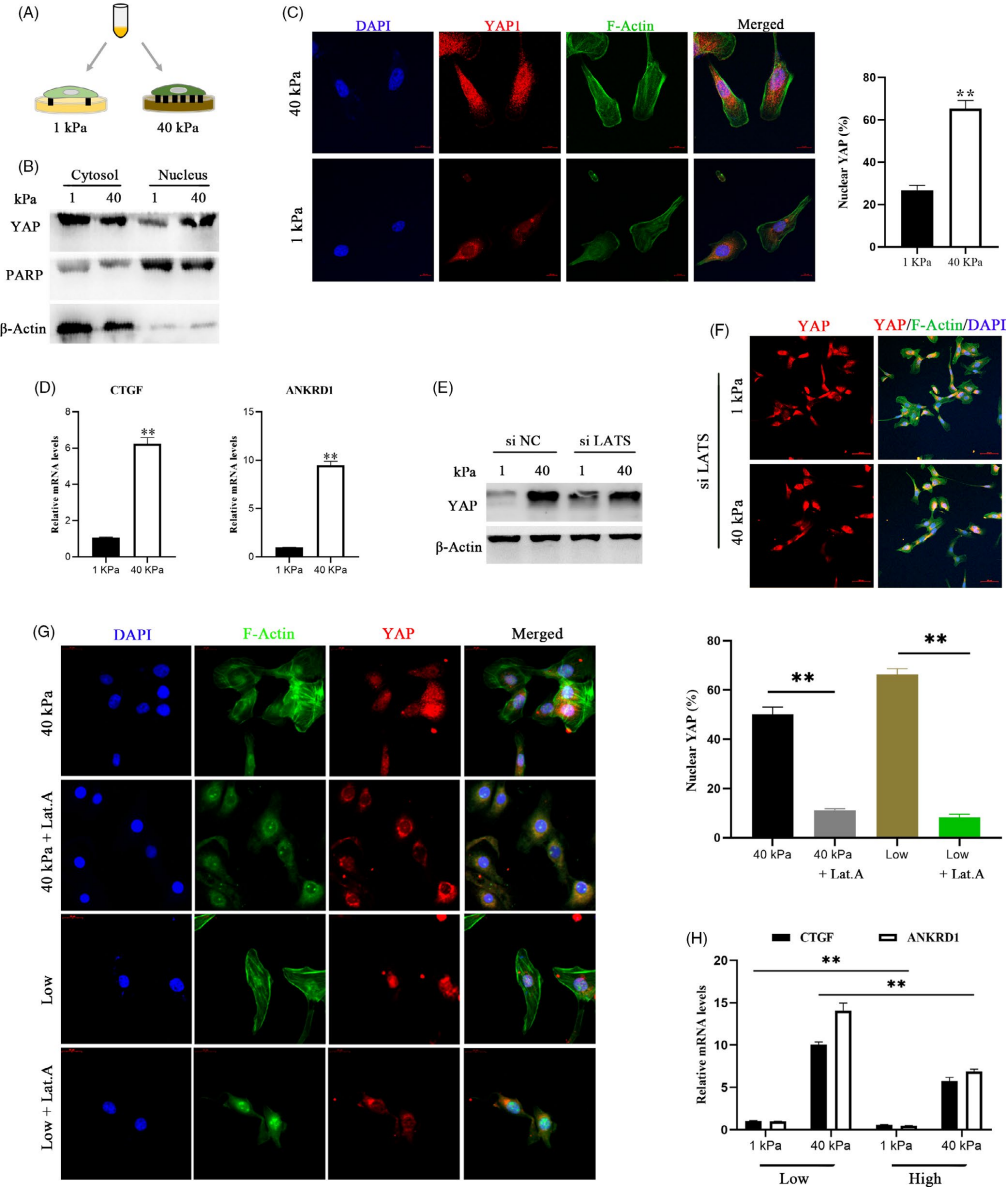
YAP is regulated by ECM stiffness independent of the Hippo pathway and requires tension of the actin cytoskeleton. a. Schematic representation of bEECs plated on hydrogels with different rigidities (40/1 kPa). B, Western blotting for YAP in nuclear and cytoplasmic protein fractions from bEECs plated on 40 kPa and 1 kPa fibronectin‐coated hydrogels for 48 hour, n = 3. C, Confocal immunofluorescence images (left, n = 2) and quantifications of nuclear and cytoplasmic subcellular localization (right, n = 15) of YAP in bEECs plated on hydrogels with different rigidities. Scale bars, 10 μm. D, RT‐ qPCR analysis of mRNA levels of CTGF and ANKRD1 in bEECs plated on hydrogels with different rigidities, n = 3. E, Protein expression of YAP in bEECs transfected with siNC or siLATS at 40/1 kPa hydrogels, n = 3. F, Representative immunofluorescence of YAP in bEECs transfected with si LATS and cultured on hydrogels, n = 2. Scale bars, 50 μm. G, Confocal immunofluorescence images (left, n = 2) and quantification of nuclear and cytoplasmic subcellular localization (right, n = 15) of YAP in bEECs plated at 40 kPa or low density. Cells were also treated with the F‐actin inhibitor latrunculin A (Lat.A, 0.5 μM) or PBS (control) for 24 hour. Scale bars, 20 μm. h. RT–qPCR of bEECs grown under low or high conditions on the indicated hydrogels, n = 3. Experiments were repeated *n* times with two biological replicates. Data are shown as the mean ± SEM. *P* values were determined by an unpaired two‐sided t test (c, d, g) and two‐way ANOVA (h). **P < .001. DAPI, blue, nuclei; F‐actin, green, cell boundaries; YAP, red. See also Figure S2d–h

To test whether YAP activity is regulated by ECM stiffness in bEECs, we evaluated YAP pathway activity in bEECs grown on fibronectin‐coated acrylamide hydrogels of varying stiffness (elastic modulus ranging from 1 to 40 kPa, matching the physiological elasticities of natural tissues). The activity of YAP in cells grown on stiff hydrogels (~40 kPa) was comparable to that of cells grown at low cell density, whereas growing cells on soft matrices (~1 kPa) inhibited YAP activity to levels comparable to those of cells grown at high cell density (Figure 3A‐D, Figure S2D). Interestingly, knockdown of LATS1/2 was not sufficient to block YAP regulation by mechanical forces in in vitro cultured cells grown on synthetic substrates (Figure 3E,F, Figure S2E). The results indicated that YAP regulation through matrix stiffening was largely independent of the Hippo pathway. Based on previous studies^30^, ^31^ and the above results (Figures 2C, 3C), we also speculated that F‐actin integrity is required for YAP to maintain sensitivity to cell density and ECM compliance. In bEECs, activation of YAP by growth at a low‐density or on a stiff hydrogel was abolished by the addition of latrunculin A, an F‐actin inhibitor (Figure 3G, Figure S2F). Moreover, plating cells at high density combined with soft matrices to inhibit YAP activity (Figure 3H, Figure S2G,H). Collectively, these observations suggest that YAP activity and subcellular localization are regulated by the Hippo signalling pathway and mechanical cues.

### 3.3 YAP/TAZ activity is regulated by IFNτ

**FIGURE 4:**
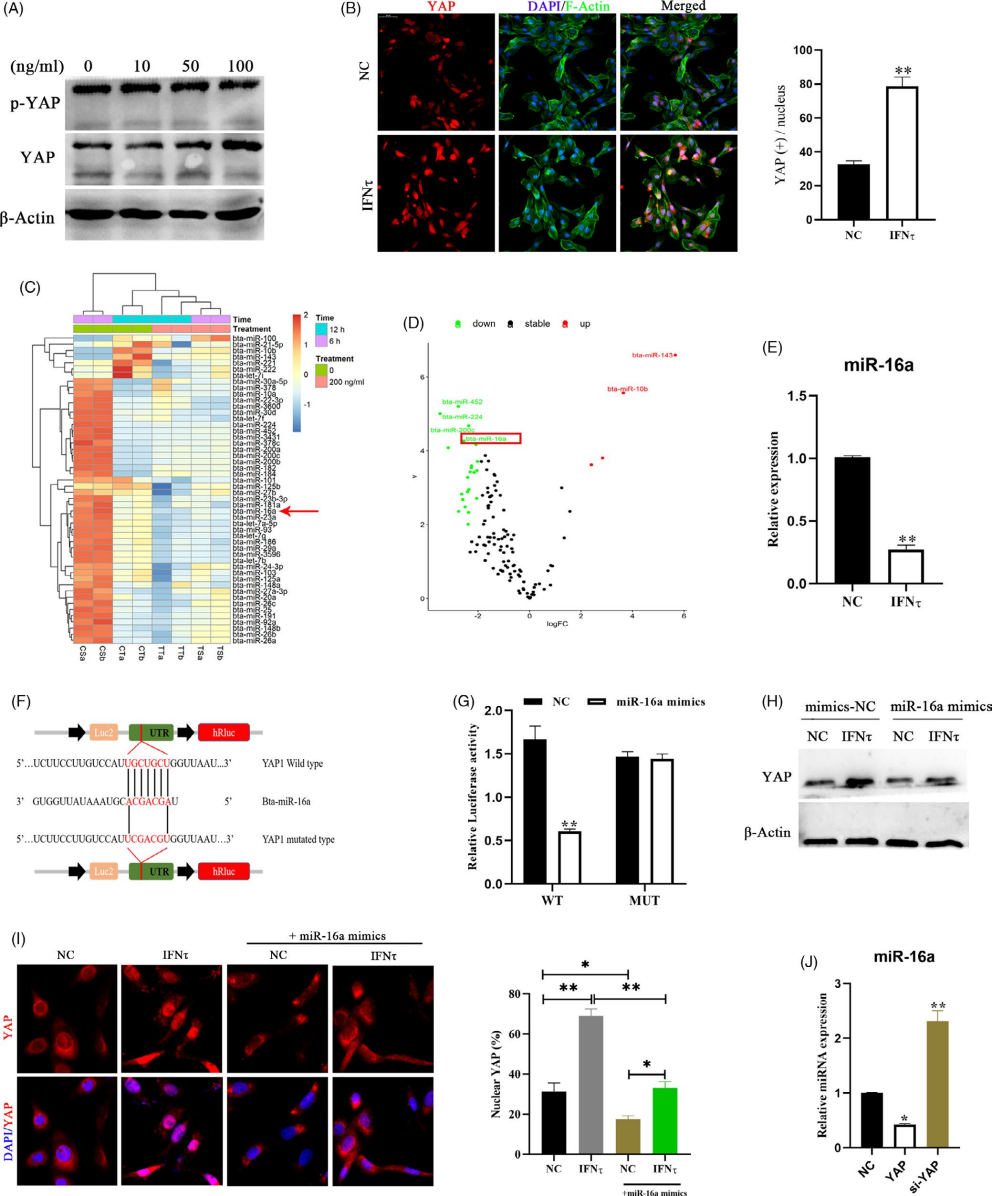
IFNτ mediates the regulation of YAP by decreasing the expression of miR‐16a. A, Protein expression levels of YAP detected in bEECs treated with 10, 50, or 100 ng/ml IFNτ for 24 hour by western blot, n = 3. B, Immunofluorescence images (left, n = 2) and quantification of YAP positive cells (right, n = 5) in bEECs treated with 100 ng/ml IFNτ. Scale bars, 50 μm. C, Heatmap showing commonly expressed miRNAs with significant expression variance. The colour scale indicates relative expression levels of miRNAs. CT and CS: PBS treatment for 12 hour and 6 hour, respectively. TS and TT: IFNτ treatment for 12 hour and 6 hour, respectively. D, Volcano plot for abnormal expression of miRNAs after 12 hour of bovine epithelial cells treated with 100 ng/ml IFNτ. Differentially expressed miRNA were exhibited a 2‐fold change in expression with an adjusted *P* value of 0.05. E, RT‐qPCR analysis of relative miR‐16a expression levels in bEECs treated with 100 ng/ml IFNτ, n = 3. F, Schematic diagram showing dual‐luciferase reporter constructs harbouring the 3′‐UTR of YAP with the putative miR‐16a‐binding site. G, Luciferase activity was measured using the dual‐luciferase reporter assay system, n = 3. H, Expression of YAP protein after treatment with the indicated regimen, n = 3. I, Confocal immunofluorescence images (n = 2) and quantification of YAP positive cells (right, n = 15) in bEECs transfected with mimics‐NC or miR‐16a mimics and treated with IFNτ (100 ng/ml) or PBS. J, Expression levels of miR‐16a in bEECs transfected with siNC, siYAP and pcDNA3.1(+) YAP were detected using RT‐qPCR, n = 3. Experiments were repeated *n* times with two biological replicates. Data are shown as the mean ± SEM. *P* values were determined by an unpaired two‐sided t test (B, E), one‐way ANOVA (I, J) and two‐way ANOVA (G). *P < .05, **P < .01. See also Figure S3

In cattle, IFNτ expression is spatiotemporally specific, and IFNτ represents an important signalling cytokine during embryo recognition.^11^ Next, we explored whether IFNτ treatment affects the transcriptional activity of YAP. We assayed two of the most strongly IFNτ regulated genes,^32^ ISG12 and MX2, by real‐time PCR to show the effect of IFNτ (Figure S3A). Our results demonstrated that both the mRNA and protein levels of YAP increased in the IFNτ treatment group and that YAP translocated into the nucleus (Figure 4A,B and Figure S3B). Mechanistically, we noticed that IFNτ did not conspicuously affect YAP phosphorylation compared to that in the control group (Figure 4A), and then we speculated that IFNτ regulates YAP expression through an abnormally expressed miRNA. Analysing data from our previous study,^32^ we observed that miRNA‐16a attenuation by IFNτ was inversely correlated with the expression levels of YAP. (Figure 4C‐E, Figure S3C). The results of the double‐luciferase reporting experiment showed that compared to the MUT YAP‐3′UTR vector groups, luciferase activities were significantly decreased after cotransfection with WT YAP‐3′UTR vectors and miR‐16a mimics, confirming that YAP is a direct target gene of miR‐16a (Figure 4F,G, Figure S3D). Similarly, we also found that miR‐16a partially blocked the activation of YAP by IFNτ (Figure 4H,I and Figure S3E‐G). Previous studies have reported that the Hippo pathway regulates miRNA biogenesis through nuclear binding of YAP and sequestration of p72 (DDX17) in cancer.^33^ Interestingly, we observed that YAP overexpression reduced miR‐16a levels in bEECs (Figure 4J and Figure S3H). Physiologically, this positive feedback mechanism allows amplification of embryo‐derived signals. Therefore, we suggest that bEECs may respond to IFNτ signals released from the conceptus by activating the miR‐16a/YAP axis.

### 3.4 YAP activation provides the physiological environment needed for early pregnancy

**FIGURE 5:**
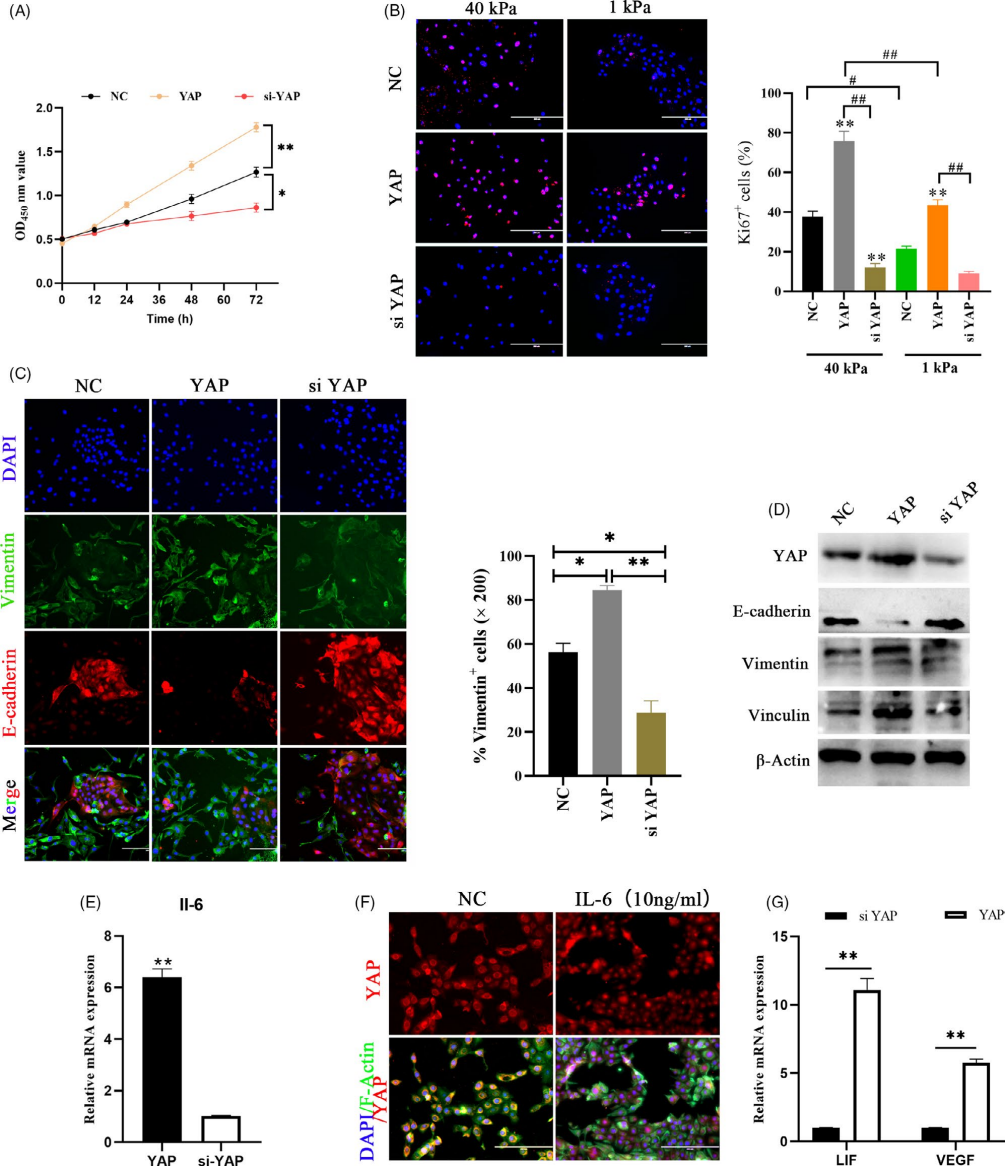
YAP activation can provides the physiological environment needed for early pregnancy. A, CCK‐8 kits were used to assess proliferation of bEECs transfected with siNC, siYAP or pcDNA3.1(+) YAP at 0, 12, 24, 48 and 72 hour. *P < .05, **P < .01. B, Immunofluorescence images (left) and quantifications of Ki67positive cells (right) in bEECs transfected with siNC, siYAP or pcDNA3.1(+) YAP at 40 kPa/1 kPa hydrogels, n = 3. Scale bars, 200 μm. **P < .01, ^#^ *P* < .05, ^##^ *P* < .01. C, Immunofluorescence images of E‐cadherin and vimentin (left, n = 2) and quantification of vimentin^+^ positive cells (right, n = 5) in bEECs transfected with YAP overexpression plasmid or siYAP at 48 hour. Scale bars, 200 μm. E‐cadherin, red; DAPI, blue; Vimentin, green. *P < .05, **P < .01. D, Protein expression levels of YAP, E‐cadherin, vimentin and vinculin were detected in bEECs, n = 3. E, RT‐qPCR analysis of relative IL‐6 expression levels in bEECs transfected with siYAP or pcDNA3.1(+) YAP, n = 3. F, Representative immunofluorescence of YAP in bEECs treated with IL‐6 (10 ng/ml) or PBS at 24 hour, n = 2. G, RT‐qPCR analysis of relative LIF and VEGF expression levels in bEECs transfected with siYAP or pcDNA3.1(+) YAP, n = 3. **P < .01. Experiments were repeated *n* times with two biological replicates. Data are shown as the mean ± SEM. *P* values were determined by an unpaired two‐sided t test (e, g) and two‐way ANOVA (A–C). See also Figure S4

Unsurprisingly, as in other cell types,^17^ YAP activation induced by mechanical or chemical signals promoted proliferation of bEECs (Figure 5A,B). This provides a theoretical basis for the growth and replacement of the maternal endometrium during pregnancy. Furthermore, we observed a decrease in E‐cadherin expression and an increase in vimentin activation in cells overexpressing YAP (Figure 5C,D, Figure S4A). This result indicates that switch‐like regulation of YAP activated by mechanical or chemical signals, induces epithelial‐mesenchymal transition (EMT) and loosens cell‐cell adhesion. Healthy pregnancy requires tightly coordinated immune responses. In early pregnancy, blastocysts attach to and invade the maternal endometrium and this progression is accompanied by evolutionarily conserved inflammatory responses, including IL‐6, IL‐1, and LIF.^34^ We observed that mechanical or chemical stimulation upregulated YAP induced IL‐6 production in bEECs and had no obvious effect on other inflammatory factors (Figure 5E, Figure S4B), which are proposed to be essential for the formation of the maternal uterine immune environment during the preimplantation period. We also found that IL‐6 positively regulates YAP activation in bEEc cells (Figure 5F), consistent with previous studies in an organ injury model.^35^, ^36^ The expression of the uterine receptive markers LIF and VEGF also exhibited a positive correlation with YAP (Figure 5G). These results indicate that YAP activation provides the conditions required for early pregnancy.

### 3.5 YAP activation is required for early pregnancy in the mouse endometrium

**FIGURE 6:**
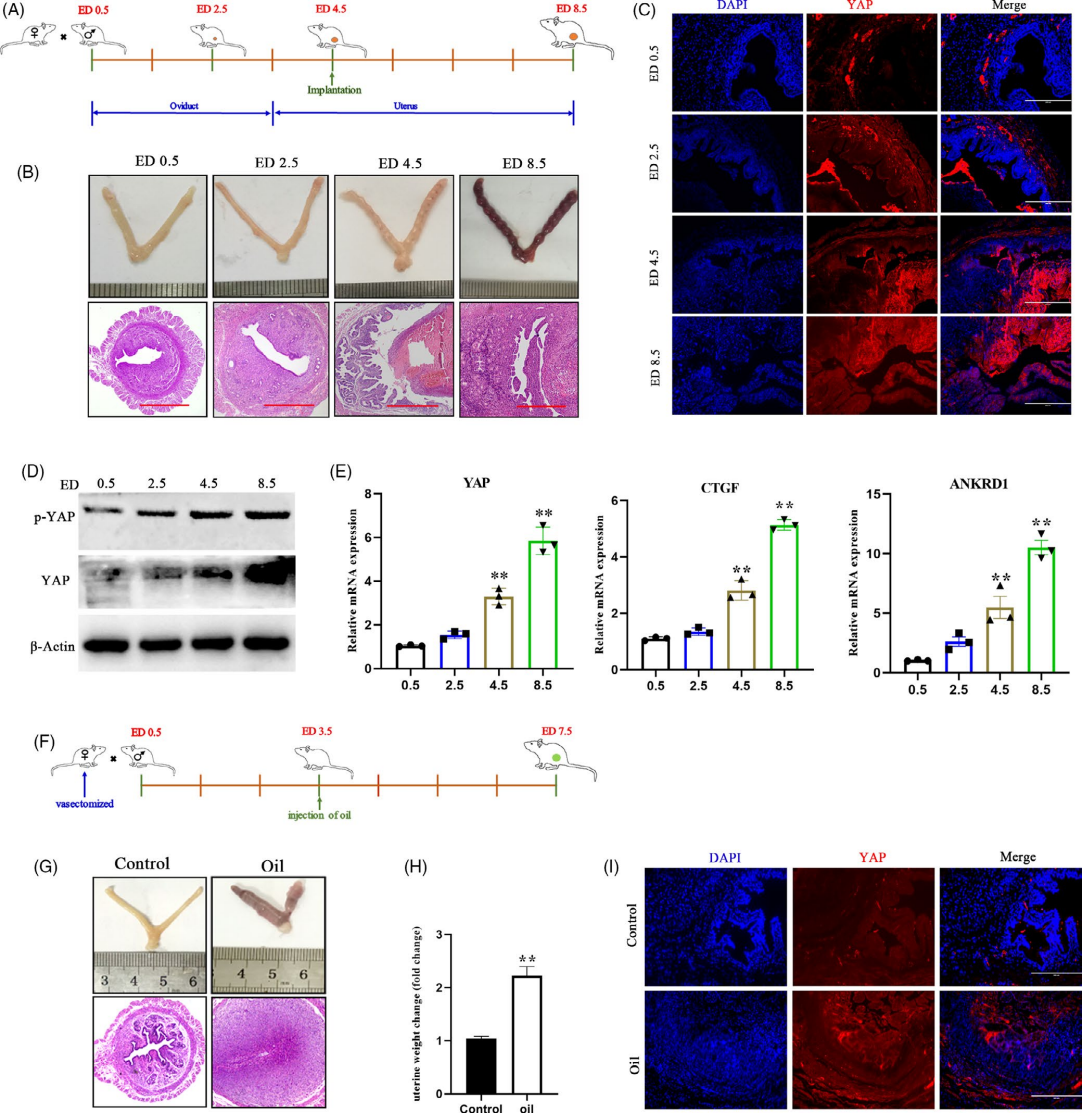
YAP is activated by mechanical and chemical cues in mouse pregnancy‐related models. A, Timeline of embryo development and euthanasia. B, Whole uterus images and uterine sections stained with H&E. Scale bars, 500 μm. C, Immunofluorescence detection of YAP endometrial sections from pregnant mice. Scale bars, 400 μm, n = 2. D, Protein expression levels of YAP and p‐YAP were detected in mouse uterine tissue, n = 3. E, RT‐qPCR analysis of YAP, CTGF and ANKRD1 expression levels, n = 3. F, Schematic outline of the establishment of pseudopregnancy mouse model. G, Schematic illustration of pseudopregnant mouse model. Whole uterus image and uterus sections stained with H&E. Scale bars, 500 μm. H, Effect of oil on uterine weight change 4 d. Data are shown as the fold change compared to controls without oil injection, n = 6. I, Immunofluorescence images of YAP showing uterine cross‐sections from pseudopregnant mice 4 d after intrauterine injection of oil, n = 2. Experiments were repeated n times with two biological replicates. Data are presented as the mean ± SEM. *P* values were determined by unpaired two‐sided t tests (h) and one‐way ANOVA (e). *P < .05, **P < .01. See also Figure S5

To further confirm the activation of YAP during pregnancy, we used a mouse pregnancy model in vivo. In agreement with the bovine in vivo data, the expression of YAP associated genes, including YAP at the protein level and CTGF and ANKRD1 at the mRNA level, was significantly augmented in the mouse model of early pregnancy (Figure 6A‐E). These results also revealed that YAP is activated before embryo implantation and without direct mechanotransduction between the embryo and maternal uterus, which indicates that chemical stimulation signals initiate YAP activation during early pregnancy (Figure 6C,D). In addition, we found that intrauterine injection with IFNτ significantly increased YAP expression in the IFNτ‐treated uterus compared to PBS‐treated control mice (Figure S5A‐E). We also observed that the expression of YAP was highest at the attachment site, which prompted us to speculate that it plays a role in mechanotransduction. Next, we tested whether blocking embryo‐derived signals would interfere with YAP activation in vivo using an oil‐induced mouse pseudopregnancy model (Figure 6F‐H, Figure S5F). Remarkably, the mechanical signals that the cell received from the uterine cavity induced activation of YAP (Figure 6I, Figure S5G). These data suggest that activated YAP is subject to regulation by both mechanical and chemical signal during early pregnancy.

### 3.6 YAP activation is necessary for early pregnancy

**FIGURE 7:**
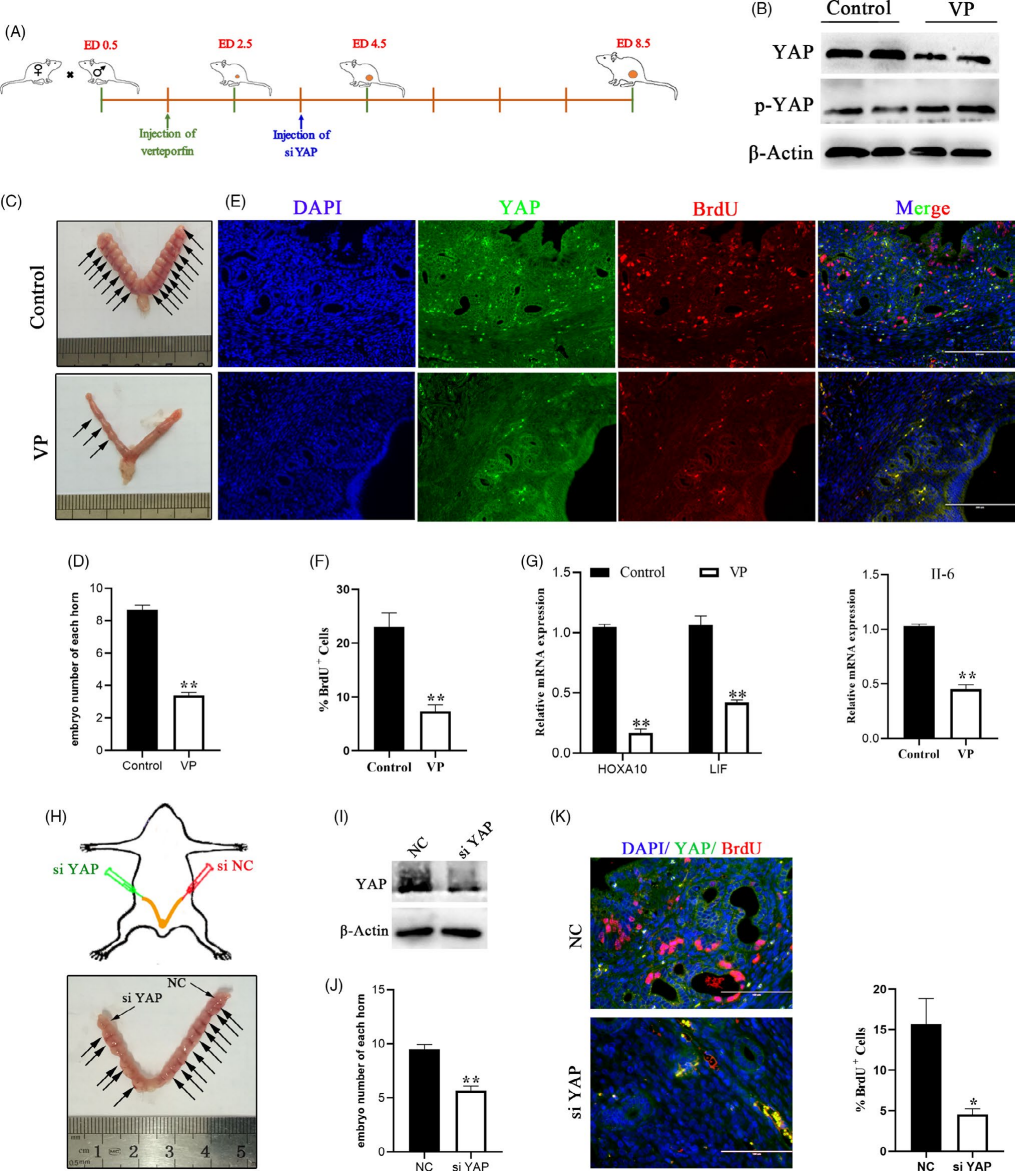
YAP is required for embryo implantation in vivo. A, Schematic illustration of intrauterine injection of siYAP or intraperitoneal injection verteporfin. B, Protein expression levels of YAP and p‐YAP were detected in mouse treated with VP or PBS (control), n = 3. C, Representative image of embryo implantation sites from the control and VP groups at ED 8.5. D, The number of implanted embryos observed in each uterine horn on day ED 8.5. Control, n = 14; VP, n = 9. E, F, Costaining of BrdU with YAP (e) and quantification of BrdU^+^ positive cells (f) in mouse endometrial sections, n = 2. Scale bars, 200 μm. G, RT‐ qPCR analysis of mRNA levels of IL‐6, HOXA 10 and LIF in the mouse uterus, n = 3. H, Effect of YAP knockdown on implantation rate in mice with an image showing implantation sites (arrows) in the control uterine horn (right, siNC) compared to the siYAP treated samples. I, Western blotting data showing uterine YAP levels after injection of siNC or siYAP, n = 3. J, The number of implanted embryos observed in each uterine horn on day ED 8.5. n = 6. K, Costaining of BrdU with YAP and quantification of positive cells (right) of BrdU^+^ in mouse endometrial sections, n = 2. Scale bars, 100 μm. Experiments were repeated *n* times with two biological replicates. Data are mean ± SEM. *P* values were determined by an unpaired two‐sided t test (D, E, I) and one‐way ANOVA (F). **P < .01

To investigate the role of YAP in the maternal uterus during early pregnancy in vivo, we administered the YAP inhibitor verteporfin to pregnant mice (Figure 7A). By western blot analysis, we clearly observed that verteporfin significantly reduced the expression of YAP (Figure 7B). Intraperitoneal injection with verteporfin reduced in the number of implanted embryos in verteporfin‐treated uterine horns compared to vehicle‐treated control horns (Figure 7C,D). Next, we analysed endometrial cell proliferation after VP treatment with bromodeoxyuridine (BrdU) and YAP double staining in mouse uterine tissue (Figure 7E). By scoring the ratio of BrdU^+^ cells versus total cells, we found that endometrial cell proliferation was decreased in the VP treatment group (Figure 7F). The expression of two implantation markers, HOXA10 and LIF, was also dramatically reduced when YAP was inhibited or knocked down (Figure 7G). In addition, inactivation of YAP leads to a reduction in the inflammatory factor IL‐6 that is required for early pregnancy (Figure 7G). To exclude a possible non‐specific effect of the YAP inhibitor on pregnant mice and embryos, we inactivated YAP by injecting independent sets of siRNAs into the uterine cavities of mice on ED 3.5 (Figure 7A and H). Similar to verteporfin, siYAP also significantly reduced the number of implanted embryos compared to that of the control treated with negative control siRNAs (Figure 7H‐K). Collectively, these results suggest that YAP is a vital factor in the maternal uterus that is necessary for successful pregnancy.

## 4 DISCUSSION

Previous studies of YAP have shown that it determines organ size and tumorigenesis, but investigation of YAP function in the uterus during early pregnancy are limited. Here we show that activation of YAP provides a suitable environment for early pregnancy in maternal endometrial tissue. Indeed, high YAP expression promoted endometrial cell proliferation, induced EMT progression and generated a small inflammatory benefit to enable the uterus to receive the embryo. Mechanistically, the expression levels of YAP are dually regulated by upstream biochemical and mechanical signals in bEECs and are partially dependent on the Hippo signalling pathway.

Although many clues have been uncovered indicating that YAP and TAZ are sensors of the structural and mechanical features of the cellular microenvironment,^37^, ^38^ the exact mechanism by which mechanical signals modulate YAP nuclear localization is not entirely clear. Mechanical inputs represent a central pillar in the control of YAP/TAZ activity, including modification of the actomyosin cytoskeleton, cell polarity, matrix stiffness and cell shape.^29^, ^30^, ^39^ In this study, we confirmed that cell density and ECM stiffness affect YAP expression and nuclear translocation, consistent with previous studies.^29^, ^30^, ^39^ However, there is controversy about whether mechanical signals regulate YAP/TAZ through the Hippo pathway. Recently, some studies have suggested that YAP regulation by stiffness and Rho is independent of the Hippo pathway,^39^, ^40^ but on the other hand, studies have demonstrated that YAP regulation by matrix stiffening involves both the actin cytoskeleton and the Hippo pathway.^30^, ^41^ Here, we showed that the effect of cell density is largely dependent on the Hippo pathway, and that the ECM yielded the opposite results. This inconsistency could be due to the form, character or magnitude of the mechanical forces, and the threshold difference needed for cells to detect the mechanical force. In addition, there is a consensus that actin is important for mechanotransduction, and the Hippo pathway contributes to remodelling both the actin cytoskeleton and the ECM.^42^

Quite a few types of soluble factors have been reported to activate YAP in mammals; these factors include LPA and oestrogen, which depend on G protein–coupled receptors (GPCRs).^43^, ^44^ In contrast, cytokines, insulin and IL‐6 act independently of GPCRs and can also modulate cell processes via the Hippo‐YAP pathway.^38^, ^45^ Our study suggests that IFNτ is a critical factor for pregnancy recognition in ruminant ungulates and activates YAP in bEECs. Moreover, we revealed that miRNA‐16a is the core downstream component of IFNτ‐mediated YAP activation. It has been reported that YAP represses miRNA biogenesis and may be involved in the widespread miRNA repression observed in cancer.^33^ This indirectly explains why we observed that YAP inhibits expression of miRNA‐16a. Interestingly, an early study demonstrated that YAP regulates IFNτ expression via the TEAD transcription factor in bovine conceptuses.^46^ Taken together, this seems to imply that there is a complex feedback network between IFNτ and YAP during early pregnancy. Future work will need to focus on revealing the relative contribution and possible collaboration of biochemical and mechanical signals between the embryo and the uterus.

Although YAP appears to be dispensable for physiological homoeostasis in several adult organs, it is critical to promote tissue repair in response to injury and serves as an oncogene in many types of tumours.^37^, ^45^ Here, we demonstrated that YAP activity is regulated by both biochemical and mechanical cues and that its activation promotes endometrial cell proliferation. Consistent with previous studies, YAP is highly expressed in cancer and correlated with cancer cell characteristics, such as proliferation, migration and apoptosis.^47^, ^48^ Although YAP contribute to repair and alleviates inflammatory damage,^15^, ^49^ we showed that YAP induced the production of the proinflammatory cytokine IL‐6. In embryos, differences in YAP expression levels and subcellular localization between internal and external cell populations lead to changes in cell fate specification during trophectoderm (TE) formation.^50^ Outer TE cells invade the endometrium and are involved in the EMT process, and our data demonstrate that overexpression of YAP upregulates of EMT‐associated transcription factor expression in bEECs. Furthermore, we used mouse pregnancy and pseudopregnancy models to reveal a crucial role for YAP activation by mechanical and chemical signals in facilitating successful pregnancy. Inhibiting the activation of YAP causes the failure of conceptus attachment to the uterine epithelium and a reduction in the number of embryos.

In summary, this study is the first to show that activation of YAP is necessary for early pregnancy in bovines because it induces the maternal uterus to provide an environment suitable for conceptus survival. Mechanistically, we suggest that YAP is a sensor for endometrial cells to respond to extracellular chemical and mechanical signals, and that different stimuli converge on YAP regulation through the IFNτ/miR‐16a/YAP or Hippo/F‐Actin/YAP pathways. Identification of this underlying mechanistic axis provides novel insights into how the maternal uterus responds to signals from the surrounding environment during early pregnancy.

## CONFLICT OF INTERESTS

The authors declare no competing financial interests.

## AUTHOR CONTRIBUTIONS

TZ and GD conceived and designed the experiments. TZ, SG, and HZ carried out the experiments. TZ, CQ, and GZ analysed the data. TZ, ZW, J.L, and GD wrote the manuscript. All authors agreed to be responsible for the content of the work.

## Supporting information

Figure S1‐S5

## Data Availability

All other data that support the findings of this study are available on request from the corresponding author. The RNA‐seq datasets analysed in this article are publicly available in the GEO database under the identifiers GSE107891, https://doi.org/10.3168/jds.2011‐5114 and https://doi.org/10.18632/oncotarget.18470.
