# Supplementary material for: Endometrial extracellular matrix rigidity and IFNτ ensure the establishment of early pregnancy through activation of YAP
Source: Cell Prolif. 2021 Jan 4;54(2):e12976. doi: 10.1111/cpr.12976 (PMC7849163; doi:10.1111/cpr.12976)
Supplement: Supplementary file 1 — Figure S1‐S5 [file CPR-54-e12976-s001.docx]

**Endometrial extracellular matrix rigidity and IFNτ ensure the establishment of early pregnancy** **through activation of YAP**

Tao Zhang^1^, Shuai Guo^1^, Han Zhou^1^, Zhimin Wu^1^, Junfeng Liu^2^, Changwei Qiu^1^, Ganzhen Deng^1^*

^1^ Department of Clinical Veterinary Medicine, College of Veterinary Medicine, Huazhong Agricultural University, Wuhan 430070, People’s Republic of China.

^2^ College of Animal Science, Tarim University, Alar, Xinjiang 843300, People's Republic of China.

*** correspondence**

Ganzhen Deng, College of Veterinary Medicine, Huazhong Agricultural University, Wuhan 430070, People’s Republic of China.

**E-mail:** 925651088@qq.com

**Contents**

Supplement Figure S1-S5

Supplement Table S1- S5

**Supplement Figure**


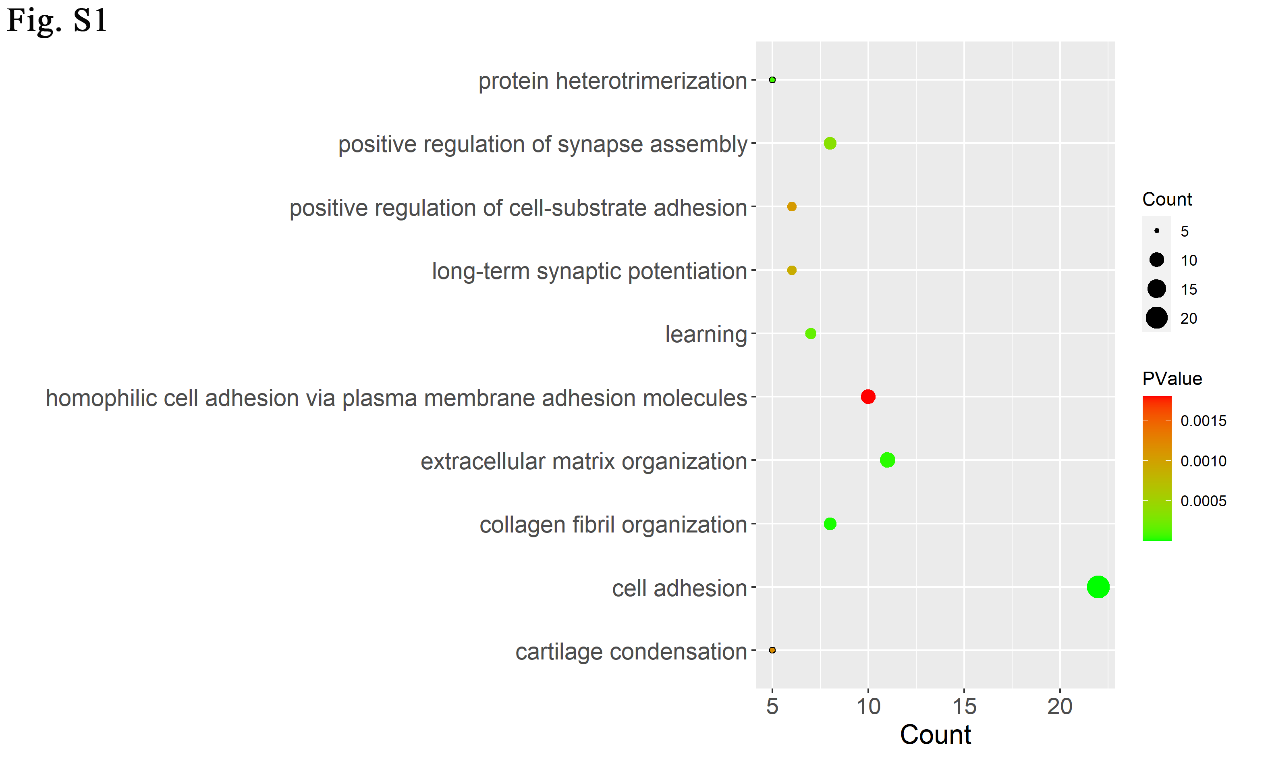


**Fig. S1.** Identification of DEGs in the pregnant bovine endometrium from the GEO dataset (GSE107891) and supplement data from a previous study (doi: 10.3168/jds.2011-5114). P< 0.01, |log FC| > 2. The top 10 GO (biological process) term of differentially expressed genes in the pregnant vs nonpregnant group.


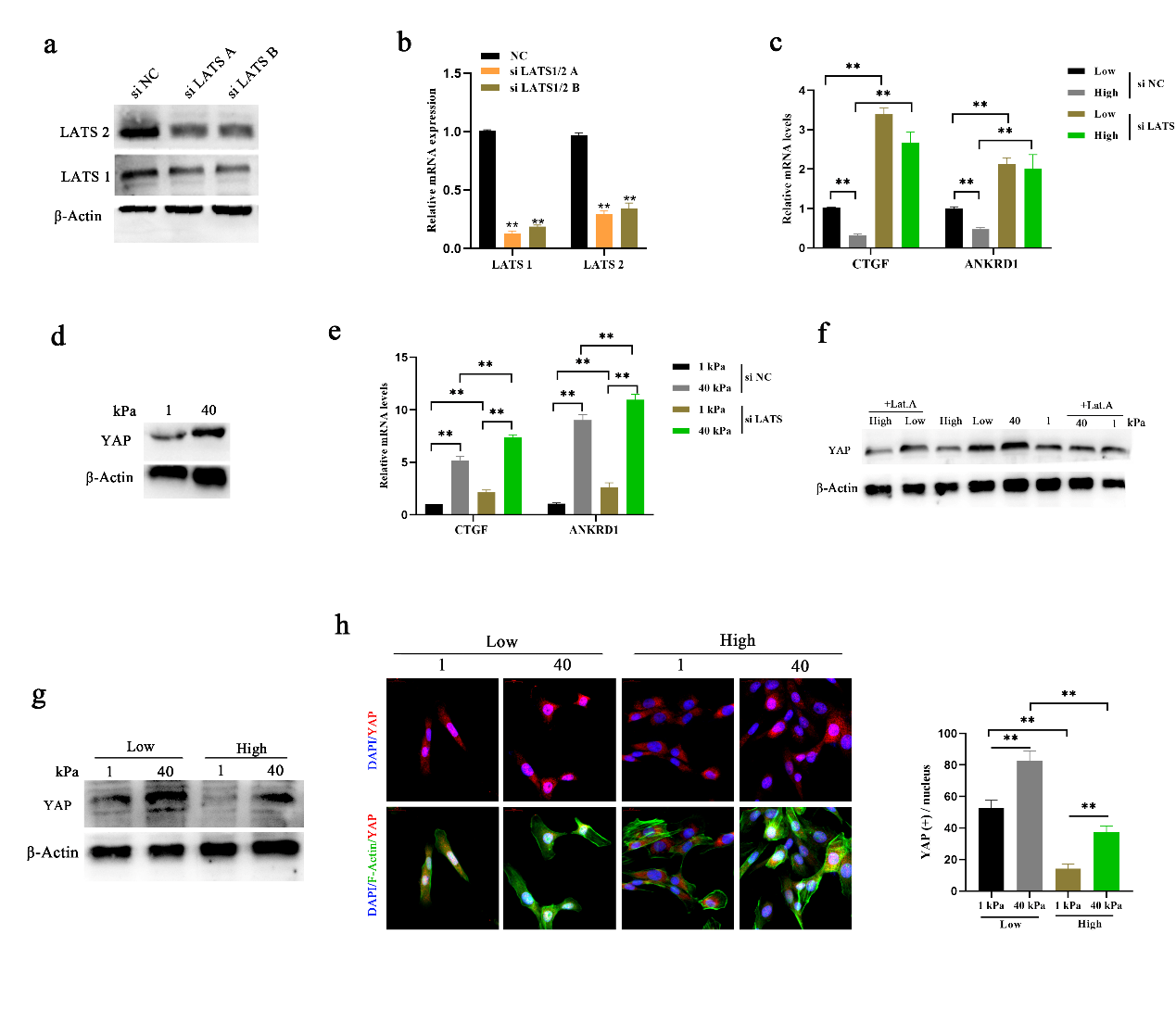


**Fig. S2. YAP is regulated by cell density and ECM stiffness. a, b.** Protein and mRNA levels of LATS1 and LATS2 were detected by immunoblots and RT-qPCR, after cotransfection with siLATS1/2(A or B). n=3. **c.** mRNA levels of CTGF and ANKRD1 in bEECs cultured at low/high density and transfected with siLATS or siNC were detected by RT-qPCR. n=3. **d.** The total protein expression in YAP of bEECs plated on 40 kPa and 1 kPa fibronectin-coated hydrogels for 48 h. n=3. **e.** mRNA levels of CTGF and ANKRD1 by RT-qPCR in bEECs transfected with siNC or siLATS at 24 h on 0 kPa/1 kPa hydrogels. **f.** Protein expression of YAP in bEECs treated with latrunculin A or PBS for 24 h at different cell densities and different ECM stiffnesses. n=3. **g, h.** Western blotting (n=3) and immunofluorescence staining (n=2) for YAP in bEECs plated on hydrogels with different rigidities and at different cell density. Experiments were repeated n times with two biological replicates. Data are presented mean ± s.e.m. P values were determined by one-way ANOVA (**b**) and two-way ANOVA (**c, e, h**). **P < 0.001. DAPI, blue, nuclei; F-actin, green, cell boundaries; YAP, red.


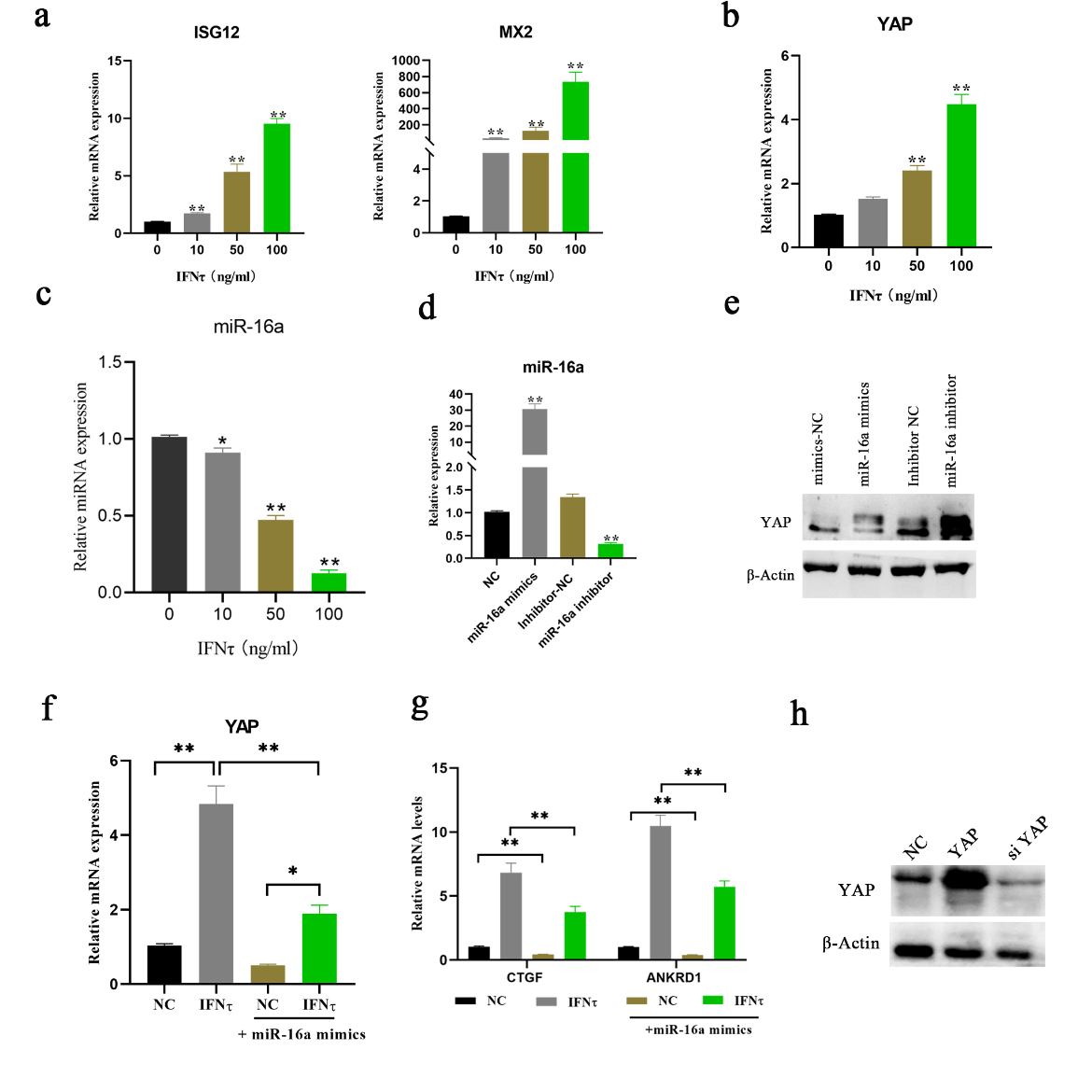


**Fig. S3 IFNτ mediates the regulation of YAP by decreasing the expression of miR-16a. a-c.** RT‐qPCR analysis of relative ISG12, MX2 (**a**), YAP (**b**) and miR‐16a (**c**) expression levels in bEECs treated with different concentrations of IFNτ. n=3. **d, e.** mRNA levels of miR-16a (**d**) and protein levels of YAP (**e**) in bEECs transfected with mimics-NC, miR-16a mimics, inhibitor-NC and miR-16a inhibitor at 24 h by RT-qPCR and western blotting, respectively. n=3. **f, g.** mRNA levels of YAP, CTGF and ANKRD1 in bEECs transfected with mimics-NC or miR-16a mimics and treated with IFNτ (100 ng/ml) or PBS were detected by RT-qPCR, n=3. **h.** Expression of the YAP protein after bEECs were transfected with si YAP or pcDNA3.1(+) YAP at 48 h was analyzed. n=3. Experiments were repeated n times with two biological replicates. Data are presented as the mean ± s.e.m. P values were determined by one-way ANOVA (**a, b, c, d**). and two- way ANOVA (**f, g**). *P < 0.05, **P < 0.01.


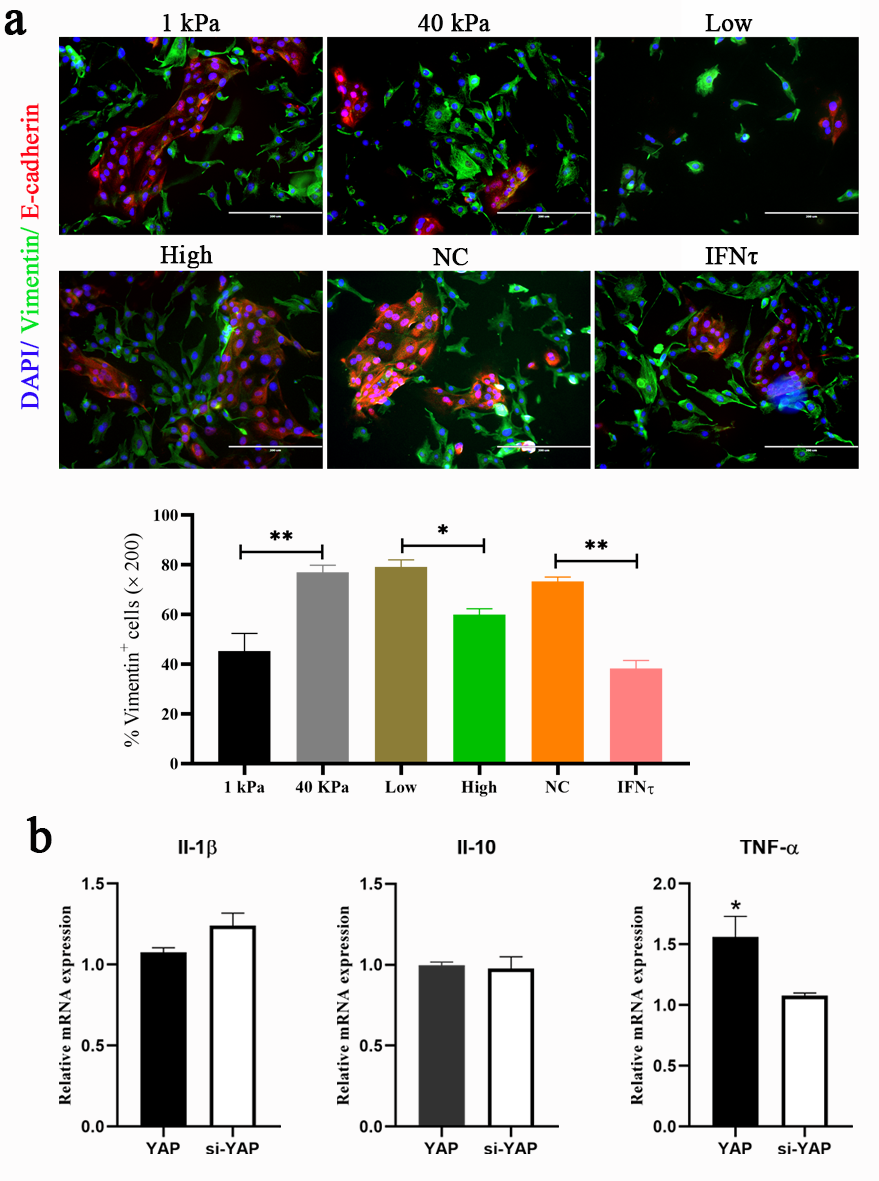


**Fig. S4 YAP activation can provide the physiological environment needed for early pregnancy. a.** Confocal immunofluorescence images of E-cadherin with vimentin (n=2) and quantification of vimentin^+^ positive (n=5) bEECs treated with the indicated regimen were analyzed, Scale bars, 200 μm. **b.** RT‐qPCR analysis of relative IL-1β, IL-10 and TNF-α expression levels in bEECs transfected with si YAP or pcDNA3.1(+) YAP. n=3, *P < 0.05, **P < 0.01. Experiments were repeated n times with two biological replicates. Data are presented as the mean ± s.e.m. P values were determined by an unpaired two-sided t-test.


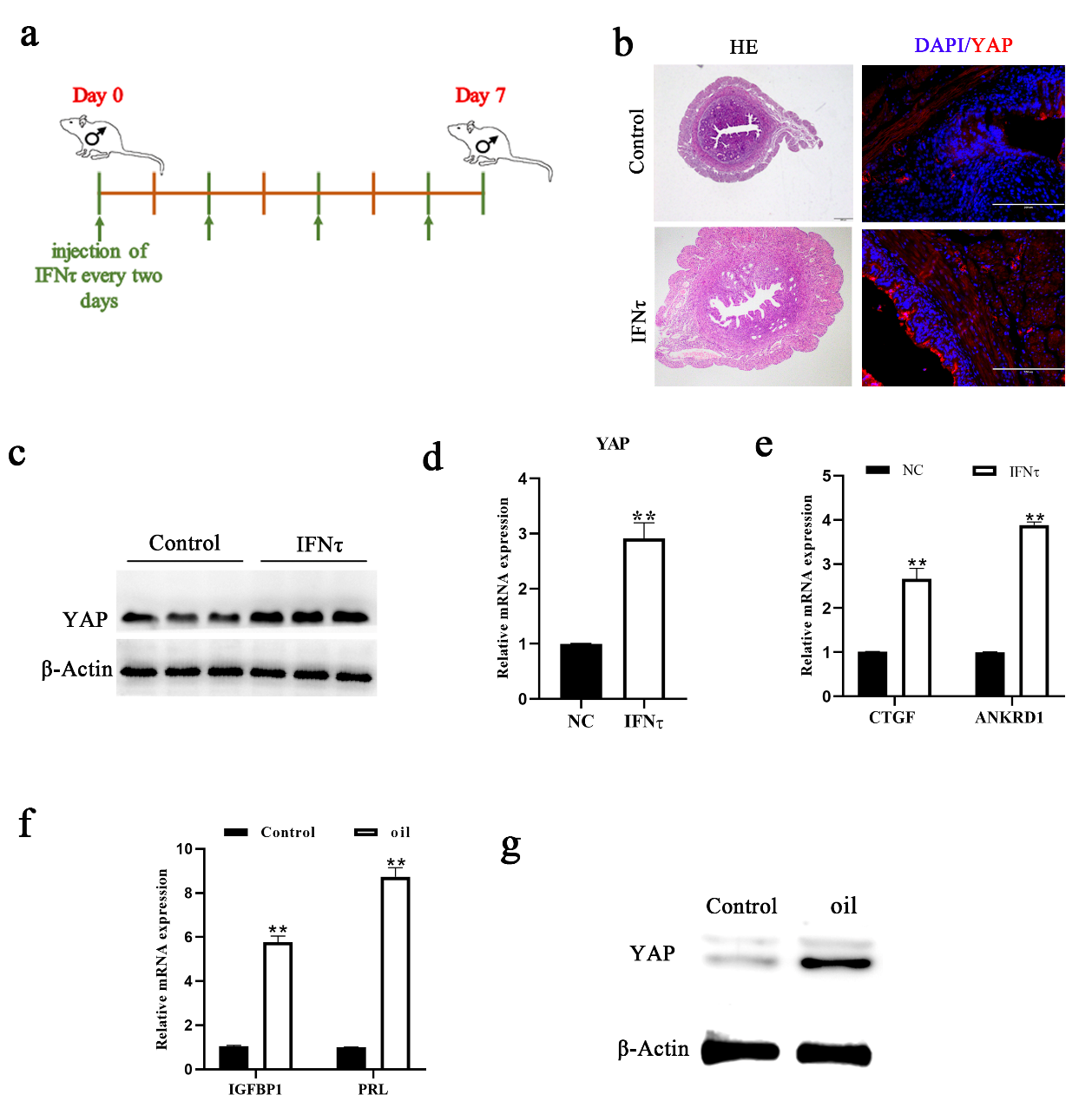


**Fig. S5 YAP is activated in mouse pregnancy-related models. a.** Timeline of IFNτ treated mice and euthanization procedures. **b.** Uterine sections stained with H&E and immunofluorescence of YAP from the control group and IFNτ group at ED 7.5. **c.** Western blotting data showing uterine YAP levels after injection of IFNτ. n = 3. **d, e.** The mRNA levels of YAP, CTGF and ANKRD1 in mouse uterus after treatment with IFNτ. n = 3. **f.** The mRNA expression levels of IGFBR1 and PRL, which are typical markers of decidualization, after pseudopregnant female mice were treated with oil. n=3. **g.** Western blotting data showing uterine YAP levels in pseudopregnant mice 7.5 d after intrauterine injection of oil. n = 3. Experiments were repeated n times with two biological replicates. Data are presented as the mean ± s.e.m. P values were determined by an unpaired two-sided t-test. **P < 0.01.

| **Table S1 miRNA primers design** | | |
| --- | --- | --- |
| miR-16a mimics | Sense | UAGCAGCACGUAAAUAUUGGUG |
|  | Antisense | CGCCAAUAUUUACGU-GCUGCUA |
| mimics-NC | Sense | UUCUCCGAACGUGUCACGUTT |
|  | Antisense | ACGUGACACGUUCGGAGAATT |
| miR-16a inhibitors | Sense | CGCCAAUAUUUACGUGCUGCUA |
| inhibotor-NC | Sense | CAGUACUUUUGUGUAGUACAA |
| si YAP | Sense | GGAGAAGUUUACUACAUAATT |
|  | Antisense | UUAUGUAGUAAACUUCUCCTT |
| si LATS 1 | Sense | GCAGUCGAAAGUGUUCAUATT |
|  | Antisense | UAUGAACACUUUCGACUGCTT |
| si LATS 2 | Sense | AAAGGCGUAUGGCGAGUAG |
|  | Antisense | GCCACGACUUAUUCUGGAA |
| si NC | Sense | UUCUCCGAACGUGUCACGUTT |
|  | Antisense | ACGUGACACGUUCGGAGAATT |

| **Table S2 miRNA primers design** | | |
| --- | --- | --- |
| U6 | ST | CGCTTCACGAATTTGCGTGTCAT |
|  | Forward | GCTTCGGCAGCACATATACTAAAAT |
|  | Reverse | CGCTTCACGAATTTGCGTGTCAT |
| bta-miR-16a | ST | CTCAACTGGTGTCGTGGAGTCGGCAATTCAGTTGAGcaccaa |
|  | Forward | GCCGAGtagcagcacgtaaata |
|  | Reverse | CTCAACTGGTGTCGTGGAGTC |

| **Table S3 oligonucleotide primers used for qPCR.** | | | |
| --- | --- | --- | --- |
| **Species** | **Gene** | **Forward** | **Reverse** |
| **bovine** | YAP | CAGAACCGTTTCCCAGACTAC | ATCAGCTCCTCTCCTTCTATGT |
|  | IL-6 | CTACCTCCAGAACGAGTATG | CAGCAGGTCAGTGTTTGTGG |
|  | IL-1β | AAAAATCCCTGGTGCTGGCT | GGGTGGGCGTATCACCTTTT |
|  | IL-10 | CTGCCTCCCACTTTCTCTTG | TCAAAGGGGCTCCCTAGTTT |
|  | TNF-α | CTCCTTCCTCCTGGTTGCAG | CACCTGGGGACTGCTCTTC |
|  | CTGF | AGCTGACCTGGAGGAGAACA | GTCTGTGCACACTCCGCAGA |
|  | ANKRD1 | AACCTGTGGATGTGCCTATG | CTGTGAAGAGCTGTCCGTTTA |
|  | ISG 12 | TGCAGAACTGCATCTCCATC | TTCATGAGGCCGTATTCCTC |
|  | MX 2 | GGTCCACCTGAACGCATATT | TCATGGCTTTCTGGACCTTATC |
|  | LIF | CAGCAACCTCATGAACCAGA | GGGCCGTGTAATAGAGGATAAAG |
|  | VEGF | CCTGATGCGGTGCGGGGGCT | TGGTGGTGGCGGCGGCTATG |
|  | GAPDH | GGTCACCAGGGCTGCTTT | CTGTGCCGTTGAACTTGC |
| **mouse** | YAP | GCGGTTGAAACAACAGGAATTA | TGAGACATCCCAGGAGAAGA |
|  | IL-6 | TTCCATCCAGTTGCCTTCTTG | CATTTCCACGATTTCCCAGAGA |
|  | IL-1β | GCAGCAGCACATCAACAAGA | GTTCATCTCGGAGCCTGTAGT |
|  | TNF-α | ACTGGCAGAAGAGGCACTC | GGCTACAGGCTTGTCACTC |
|  | CTGF | CCAATGACAACGCCTCCTG | TGGTGCAGCCAGAAAGCTC |
|  | ANKRD1 | GTGTAGCACCAGATCCATCG | CGGTGAGACTGAACCGCTAT |
|  | LIF | AGCGACATCTAACCACAGAAG | AAAGGCTTCCAGTAGACGATTAG |
|  | IGFBP1 | TATGATGGCTCGAAGGCTCT | CCATTCTTGTTGCAGTTTGG |
|  | PRL | TCTCGCCTTTCTGCTTATTATAAC | CGATTCGGCACTTCAGGAGCTT |
|  | HOXA 10 | GCTGGAGAAGGAGTTTCTGTT | CAGTTTCATCCTGCGGTTCT |
|  | GAPDH | CCATGTTCGTCATGGGTGTG | CAGGGGTGCTAAGCAGTTGG |

| **Table S4. Information on antibodies used in the study** | | | | |
| --- | --- | --- | --- | --- |
| **Antibodies** | Catalogs | **Dilution** | **Species** | **Manufacturer** |
| β-actin | #3700 | 1:2000 (WB) | mouse | Cell Signaling Technology |
| Anti-rabbit IgG | #5127 | 1:2000 (WB) | mouse | Cell Signaling Technology |
| Anti-mouse IgG | #7056 | 1:2000 (WB) | Goat | Cell Signaling Technology |
| CK18 | GB11232 | 1:200 (IF) | Rabbit | Servicebio |
| Vimentin | GB12192 | 1:1000(WB),1:250 (IF) | mouse | Servicebio |
| Cy3 anti-rabbit IgG | GB21303 | 1:100 (IF) | Goat | Servicebio |
| Cy3 anti-mouse IgG | GB21301 | 1:100 (IF) | Goat | Servicebio |
| FITC anti-mouse IgG | GB22301 | 1:50 (IF) | Goat | Servicebio |
| FITC anti-rabbit IgG | GB22303 | 1:50 (IF) | Goat | Servicebio |
| YAP | ab81183 | 1:1000 (WB), 1:100 (IF), 1:100 (IHC) | Rabbit | abcam |
| PARP | ab32064 | 1:5000 (WB) | Rabbit | abcam |
| Ki 67 | ab15580 | 1:100 (IF) | Rabbit | abcam |
| YAP1 | bs-3605R | 1:1000 (WB), 1:250 (IF) | Rabbit | Bioss |
| Phospho-YAP1 (Tyr407) | bs-3476R | 1:1000 (WB) | Rabbit | Bioss |
| Foxa2 | ab60721 | 1:200 (IF) | rabbit | abcam |
| YAP | AF6328 | 1:1000 (WB), 1:200 (IF) | Rabbit | Affinity Biosciences |
| Phospho-YAP (Ser127) | AF3328 | 1:1000 (WB) | Rabbit | Affinity Biosciences |
| LATS1 | DF7517 | 1:2000 (WB) | Rabbit | Affinity Biosciences |
| LATS2 | AF7939 | 1:1000 (WB) | Rabbit | Affinity Biosciences |
| E-cadherin | sc-8426 | 1:1000 (WB), 1:200 (IF) | mouse | Santa Cruz Biotechnology |
| EpCAM | sc-66020 | 1:50(IF) | mouse | Santa Cruz Biotechnology |
| YAP | sc-376830 | 1:1000 (WB), 1:50 (IF), 1:50 (IHC) | mouse | Santa Cruz Biotechnology |

| **Table S5. Summary of statistical test and exact P-values for each specific figure (If P-value was <0.0001 the exact value is not given by the analysis software)** | | | | | |
| --- | --- | --- | --- | --- | --- |
| **Figure** | **Name** | **Compared pairs** | **P-value** | **Test used** | **significance** |
| Fig 1f | YAP | non-pregnancy VS pregnancy | 0.0008 | Unpaired T-test | ** |
| Fig 1g | CTGF | non-pregnancy VS pregnancy | <0.0001 | Unpaired T-test | ** |
|  | ANKRD1 | non-pregnancy VS pregnancy | <0.0001 | Unpaired T-test | ** |
| Fig 1h | YAP^+/+^Positive cell | non-pregnancy VS pregnancy | 0.0068 | Unpaired T-test | ** |
| Fig 1i | Nuclear YAP^+/+^ | non-pregnancy VS pregnancy | <0.0001 | Unpaired T-test | ** |
| Fig 2d | Nuclear YAP^+/+^ | Low density VS High density | 0.0045 | Unpaired T-test | ** |
| Fig 2f | CTGF | Low density VS High density | 0.00037 | Unpaired T-test | ** |
|  | ANKRD1 | Low density VS High density | 0.00015 | Unpaired T-test | ** |
| Fig 3c | Nuclear YAP^+/+^ | 1 kPa VS 40 kPa | 0.0009 | Unpaired T-test | ** |
| Fig 3d | CTGF | 1 kPa VS 40 kPa | <0.0001 | Unpaired T-test | ** |
|  | ANKRD1 | 1 kPa VS 40 kPa | <0.0001 | Unpaired T-test | ** |
| Fig 3g | Nuclear YAP^+/+^ | 40 kPa VS 40 kPa + Lat. A | <0.0001 | Unpaired T-test | ** |
|  |  | Low VS Low + Lat. A | <0.0001 | Unpaired T-test | ** |
| Fig 3h | CTGF | Low + 1 kPa VS High + 1 kPa | <0.0001 | Two-way ANOVA | ** |
|  |  | Low + 40 kPa VS High + 40 kPa | <0.0001 | Two-way ANOVA | ** |
|  | ANKRD1 | Low + 1 kPa VS High + 1 kPa | <0.0001 | Two-way ANOVA | ** |
|  |  | Low + 40 kPa VS High + 40 kPa | <0.0001 | Two-way ANOVA | ** |
| Fig 4b | Nuclear YAP^+/+^ | NC VS IFNτ 100 ng/ml | 0.0014 | Unpaired T-test | ** |
| Fig 4e | miR-16a | IFNτ 0 ng/ml VS 100 ng/ml | <0.0001 | One-way ANOVA | ** |
| Fig 4g | Luciferase activity | NC +WT VS miR-16a mimics + WT | 0.0005 | Two-way ANOVA | ** |
|  |  | NC +MUT VS miR-16a mimics + MUT | 0.6012 | Two-way ANOVA | ns |
| Fig 4i | Nuclear YAP^+/+^ | MiRNA NC: NC VS IFNτ 100 ng/ml | 0.0002 | One-way ANOVA | ** |
|  |  | MiR16a mimics: NC VS IFNτ 100 ng/ml | 0.0308 | One-way ANOVA | * |
|  |  | miRNA NC: NC VS miR-16a mimics: NC | 0.0350 | One-way ANOVA | * |
|  |  | miRNA NC: IFNτ VS miR-16a mimics: IFNτ | 0.0002 | One-way ANOVA | ** |
| Fig 4j | miR-16a | NC VS YAP | 0.0186 | One-way ANOVA | * |
|  |  | NC VS si YAP | 0.0003 | One-way ANOVA | ** |
| Fig 5a | proliferation | NC vs. YAP | 0.0061 | Two-way ANOVA | ** |
|  |  | NC vs. si-YAP | 0.0142 | Two-way ANOVA | * |
| Fig 5b | Ki 67 ^+/+^ positive cell | 40 kPa: NC vs. YAP | <0.0001 | One-way ANOVA | ** |
|  |  | 40 kPa: NC vs. si YAP | 0.0008 | two-way ANOVA | ** |
|  |  | 1 kPa: NC vs. YAP | 0.0023 | two-way ANOVA | ** |
|  |  | 1 kPa: NC vs. si YAP | 0.0904 | two-way ANOVA | ns |
|  |  | 40 kPa + NC VS 1 kPa + NC | 0.0201 | two-way ANOVA | **#** |
|  |  | 40 kPa + YAP VS 1 kPa + YAP | <0.0001 | two-way ANOVA | **##** |
|  |  | 40 kPa + si YAP VS 1 kPa + si YAP | 0.9681 | two-way ANOVA | ns |
| Fig 5c | % Vimentin^+^ cells | NC vs. YAP | 0.0335 | two-way ANOVA | * |
|  |  | NC vs. si YAP | 0.0373 | two-way ANOVA | * |
|  |  | YAP vs. si YAP | 0.0030 | two-way ANOVA | ** |
| Fig 5e | IL-6 | si YAP VS YAP | <0.0001 | Unpaired T-test | ** |
| Fig 5g | LIF | si YAP VS YAP | <0.0001 | Unpaired T-test | ** |
|  | VEGF | si YAP VS YAP | <0.0001 | Unpaired T-test | ** |
| Fig 6e | YAP | ED 0.5 vs. ED 2.5 | 0.3064 | One-way ANOVA | ns |
|  |  | ED 0.5 vs. ED 4.5 | 0.0002 | One-way ANOVA | ** |
|  |  | ED 0.5 vs. ED 8.5 | <0.0001 | One-way ANOVA | ** |
|  | CTGF | ED 0.5 vs. ED 2.5 | 0.3761 | One-way ANOVA | ns |
|  |  | ED 0.5 vs. ED 4.5 | <0.0001 | One-way ANOVA | ** |
|  |  | ED 0.5 vs. ED 8.5 | <0.0001 | One-way ANOVA | ** |
|  | ANKRD1 | ED 0.5 vs. ED 2.5 | 0.0929 | One-way ANOVA | ns |
|  |  | ED 0.5 vs. ED 4.5 | 0.0001 | One-way ANOVA | ** |
|  |  | ED 0.5 vs. ED 8.5 | <0.0001 | One-way ANOVA | ** |
| Fig 6h | weight | Control VS Oil | 0.0027 | Unpaired T-test | ** |
| Fig 7d | Embryo number | Control VS VP | <0.0001 | Unpaired T-test | ** |
| Fig 7f | % BrdU^+^ cells | Control VS VP | 0.0057 | Unpaired T-test | ** |
| Fig 7g | Hoxa 10 | Control VS VP | <0.0001 | Two-way ANOVA | ** |
|  | LIF | Control VS VP | 0.0003 | Two-way ANOVA | ** |
|  | II-6 | Control VS VP | 0.0002 | Unpaired T-test | ** |
| Fig 7j | Embryo number | NC VS si YAP | <0.0001 | Unpaired T-test | ** |
| Fig 7k | % BrdU^+^ cells | NC VS si YAP | 0.0268 | Unpaired T-test | * |
